# Incense Aerosol-Induced Neurotoxicity Disrupts α‑Synuclein Homeostasis in a Cellular Parkinson’s Disease Model, Distinct from Cigarette Aerosols

**DOI:** 10.1021/acs.chemrestox.6c00091

**Published:** 2026-03-30

**Authors:** Yi-En Tseng, Ming-Chu Teng, Yu-Siou Huang, Chia-Hsuan Pan, Yuan-Pin Chang, Chia C. Wang, Hsiu-Fang Fan

**Affiliations:** † Institute of Medical Science and Technology, 34874 National Sun Yat-sen University, Kaohsiung 804, Taiwan; ‡ Department of Chemistry, National Sun Yat-sen University, Kaohsiung 804, Taiwan; § Aerosol Science Research Center, National Sun Yat-sen University, Kaohsiung 804, Taiwan

## Abstract

Incense burning is a widespread indoor combustion practice, yet its neurotoxic potential and impact on α-synuclein (α-Syn) proteostasis remain poorly defined. Using SH-SY5Y cells overexpressing α-Syn as a cellular Parkinson’s disease model, we exposed cells to size-fractionated incense aerosol extracts (IAE) prepared as organic-phase (OP) or water-soluble phase (WP). α-Syn overexpression augmented vulnerability to IAE, producing greater losses in viability and pronounced increases in intracellular hydrogen peroxide (H_2_O_2_), mitochondrial membrane potential depolarization, and engagement of programmed cell-death pathways. Live-cell fluorescence cross-correlation spectroscopy (FCCS) revealed that both OP-IAE and WP-IAE shifted α-Syn from oligomeric to monomeric states in the cytosol, indicating disruption of oligomerization equilibrium. Antioxidant intervention revealed mechanistic differences compared with other indoor air pollutants, cigarette smoke. OP-IAE-induced cytotoxicity cannot be mitigated by *N*-acetylcysteine (NAC) or rutin, whereas WP-IAE-induced toxicity was partially attenuated, with NAC surpassing rutin. By contrast, for cigarette aerosol extracts (CAE), both OP- and WP-CAEs were robustly rescued by NAC and, to a lesser extent, rutin. Together, these results indicate that incense aerosols, particularly OP-IAE, engage reactive oxygen species (ROS)-linked mitochondrial injury and programmed cell-death pathways while uniquely driving α-Syn monomerization, while exhibiting relative resistance to classical antioxidant intervention compared with cigarette aerosols. This work points out incense smoke as a distinct indoor neurotoxicant with implications for α-Syn homeostasis and Parkinsonian risk in exposed populations.

## Graphical abstract

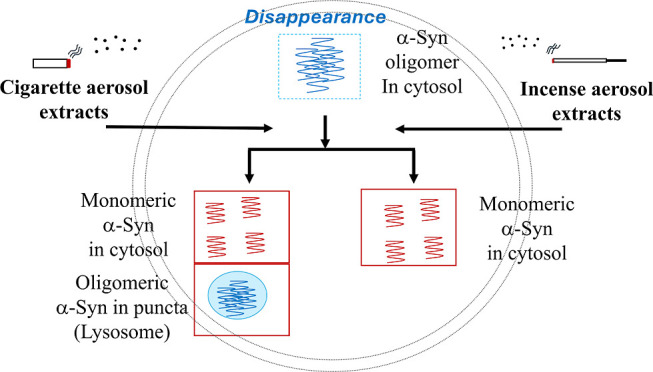

## 1 Introduction

Particulate matter (PM) is a complex mixture of solid particles and liquid droplets suspended in air. Its size distribution, chemical composition, and morphology vary with source and formation conditions (e.g., flow regime, temperature). PM originates from both natural processes (e.g., wildfires, weathered or windblown dust) and anthropogenic activities (e.g., vehicular emissions, industrial combustion).^1^ Typical constituents include inorganic ions (e.g., sulfate, nitrate, ammonium, sodium, potassium, chloride), metals (e.g., cadmium, copper, nickel, vanadium, zinc), organic and elemental carbon, polycyclic aromatic hydrocarbons (PAHs), and biological components (e.g., pollen, endotoxins, fungi, bacteria).^1–3^ PM is a major driver of air-pollution–related health burden and is strongly associated with increased premature mortality and morbidity,^4–7^ as well as higher incidence of respiratory,^8–10^ cardiovascular,^11^ and kidney diseases.^12^ Moreover, exposure to suspended PM, such as vehicular exhaust or urban pollution, has been shown to induce oxidative stress, DNA damage, neuroinflammation, and elevations of pro-inflammatory cytokines in rat brains, indicative of neuroinflammatory injury.^13,14^ These effects have been further linked to the progression of neurodegenerative diseases.^15–17^

It has been reported that individuals spend approximately 87% of their time indoors,^18^ which increases the duration of exposure to indoor air pollutants. Indoor PM_2_._5_ concentrations can exceed outdoor levels, particularly during combustion-related activities such as environmental tobacco smoke and incense burning, both recognized as major contributors to indoor air pollution.^19–22^ Incense burning is a widespread practice in many Asian regions due to its use in religious rituals, and it is also employed as a fragrance source in aromatherapy. Previous research has demonstrated that incense burning produces a significant amount of PM, with an average emission rate exceeding that of cigarette smoke.^23–25^ Notably, more than 80% of incense PM is < 0.18 μm in diameter, placing the majority of emissions in the ultrafine range.^26^ Such particles can penetrate deeply into the lungs, translocate into systemic circulation, and deposit within sensitive organs, including the brain.^27,28^ In addition to PM, the combustion process generates nitrogen dioxide, sulfur dioxide, formaldehyde, benzene, PAHs, and other volatile organic compounds, many of which have been identified as carcinogenic.^29,30^ Chronic exposure to incense smoke, therefore, results in the inhalation of hazardous substances, contributing to a wide spectrum of adverse health effects, including respiratory diseases, cognitive decline, cardiovascular disorders, and increased risks of malignancies such as lung cancer and brain tumors.^31,32^

Several epidemiological studies highlight neurotoxic effects of incense exposure across developmental stages: a case–control study in Hong Kong reported impaired cognition and motor coordination in incense users;^33^ prenatal exposure has been linked to early hyperactivity in children;^34^ and a Taiwanese cohort associated incense burning with delayed infant motor development.^35^ Children chronically exposed to incense smoke also exhibit executive function deficits, with implications for future cognitive health.^36^ Notably, incense smoke has been implicated as a potential risk factor for neurodegenerative diseases such as Alzheimer’s disease and Parkinson’s disease (PD), through mechanisms involving oxidative stress and protein aggregation.^37^ Consistent with this, a recent study demonstrated that chronic exposure to air pollutants—including fine and ultrafine particles—promotes Lewy body pathology and accelerates dementia progression, underscoring a direct mechanistic link between inhaled aerosols and α-synuclein (α-syn)–related neurodegeneration.^17^ These findings strongly suggest that incense smoke, by producing abundant ultrafine particles enriched with reactive toxicants, may contribute to both developmental neurotoxicity and age-related neurodegenerative diseases.

Recently, we found that cells treated with cigarette aerosol extracts (CAE)—where “aerosol” refers to suspended solid particles and liquid droplets in air—exhibited increased oxidative stress, mitochondrial dysfunction, and elevated levels of apoptosis, pyroptosis, and autophagy, ultimately leading to enhanced cell death.^38^ Moreover, α-syn overexpression exacerbated CAE-induced cytotoxicity, including increased reactive oxygen species (ROS) production and mitochondrial dysfunction, leading to significant cell death. Moreover, CAE exposure disrupted autophagy and altered the equilibrium of α-Syn oligomers, forming puncta localized in lysosomes.^39^ These observations imply that there is a link between cigarette aerosol exposure and the progression of neuro dysfunction. Given that incense burning is another major source of indoor aerosols, we have collected incense aerosol extracts (IAEs) from three widely used formulations—sandalwood-dominant (type A), agarwood-dominant (type B), and binchotan charcoal-dominant (type C)—and investigated the cytotoxicity in three different cell lines.^26^ It has been reported IAE derived from particles <0.18 μm exhibit the greatest biological potency, including intracellular reactive oxygen species (ROS) accumulation, mitochondrial dysfunction, autophagy activation and evaluated programmed cell death (PCD) across multiple cell types.^26^ Among three investigated incense types, type A incense exhibits the highest cytotoxicity, while SH-SY5Y cells are the most susceptible. Building on these findings, the current study explores the potential contribution of IAE to PD–related neurotoxicity. IAEs exhibiting the highest toxicity were further evaluated for synergistic effect with α-Syn in SH-SY5Y cells using protocols established in our prior CAE studies.^26,39^ Similar to CAE, α-Syn overexpression exacerbate IAE-induced ROS accumulation, mitochondrial dysfunction, and PCD. Moreover, disruption in the oligomerization equilibrium of α-Syn, driving a shift toward monomeric forms, is observed after IAEs treatment. These findings indicate that incense aerosol, similar to cigarette aerosol, poses a neurotoxic risk with implications for neurodegenerative disease pathways.^39^

In our previous work, we identified rutin, a plant-derived flavonoid, as a cytoprotective agent in CAE-treated A549, HEK293T, and SH-SY5Y cells.^38^ However, its ability to rescue cytotoxicity has not yet been investigated in SH-SY5Y cells overexpressing α-Syn. Moreover, *N*-acetylcysteine (NAC) has been shown to possess neuroprotective properties and to reduce α-Syn-induced neurotoxicity.^40–42^ Therefore, we selected the most cytotoxic IAE among the three investigated incenses for direct comparison with CAE in testing NAC and rutin as potential interventions. Both NAC and rutin alleviated oxidative stress induced by IAE and CAE. However, significant cytoprotective effects were observed only for CAE-induced toxicity, as NAC and rutin partially restored the equilibrium of α-syn oligomerization. A modest rescue effect was observed in WP-IAE–treated cells, but not in OP-IAE–treated cells. Our findings highlight incense smoke as an overlooked environmental neurotoxicant and underscore the need for further exploration of environmental risk factors and development of targeted neuroprotective strategies.

## 2 Materials and Methods

### 2.1 DNA Constructs for the Live-Cell Studies

The DNA constructs used were generated in previous studies and used here following the same protocol for cellular imaging application.^26,39,43^

### 2.2 Incense Aerosol Collection and its Extract Preparation

Three types of incense were studied¶: type A (sandalwood-dominant) and Type B (agarwood-dominant) incenses, both manufactured in Taiwan, and type C (binchotan charcoal-dominant) incense, produced in Japan.^26^ Aerosol collection and extract preparation followed our previously described protocols.^26,38,39^ Size-fractionated incense aerosols were obtained using the following cutoffs: Fraction I (>0.56 μm), Fraction II (0.56–0.18 μm), Fraction III (0.18–0.10 μm), and Fraction IV (<0.10 μm). Sandalwood-dominant incenses, exhibiting the highest cytotoxic among three investigated incenses in previous work,^26^ were further evaluated for synergistic effect with α-Syn in SH-SY5Y cells in this work and directly compare its influence to cigarette aerosol in following antioxidant experiments.

### 2.3 Cell Culture and Transfection

SH-SY5Y cells were cultured in high-glucose Dulbecco’s modified Eagle’s medium (DMEM; Gibco, 11965–084), supplemented with 1% penicillin/streptomycin (Gibco, 15140–122) and 10% fetal bovine serum (FBS; Corning, 35–010-CV). Cells were maintained at 37 °C in a humidified incubator with 95% air and 5% CO_2_. For assessments of cell viability, ROS production, mitochondrial integrity, caspase-1/3 activity, and autophagy, SH-SY5Y cells were transfected with 0.2 μg ∼1.0 μg α-Syn using TurboFectTM (Thermo Fisher, R0531) according to the manufacturer’s instructions, in the presence or absence of aerosol extracts (IAE and CAE as indicated). In our previously published study, calibration of endogenous Western blot signals indicated that endogenous α-syn levels in SH-SY5Y cells correspond to an effective transfection with <0.5 μg α-Syn cDNA under our experimental conditions.^43^ For fluorescence correlation spectroscopy (FCS) studies, SH-SY5Y cells were seeded at a density of 4.5 × 10^5^ cells per plate and transfected with 1.0 μg eGFP−α-Syn and 1.0 μg mApple−α-Syn constructs for 24–48 h using TurboFectTM (Thermo Fisher, R0531), with or without aerosol extracts. Optimal working concentrations (20 μg/mL for OP-IAE, 200 μg/mL for WP-IAE) were determined by transfection efficiency tests (Supporting Information Figure S1). Under these conditions, the transfection efficiency was approximately 50%–30%, with a surface coverage of ∼80% (Supporting Information Figure S1). This experimental design yields an overall α-syn expression level approximately three-to five-fold higher than the estimated endogenous level and was selected to model a PD–relevant cellular condition characterized by elevated α-syn burden.^43^ Before confocal imaging and FCS data acquisition, the medium was replaced with phenol red-free DMEM (Gibco, 31053–028). During data acquisition, cells were kept at 37 °C in a microincubator (Warner, DH-40iL) under 95% air and 5% CO_2_.

### 2.4 Cell Viability Assay

SH-SY5Y cells, with or without α-Syn overexpression, were seeded into 96-well plates (α-Plus, 116196–1SS) at 3.5 × 10^4^ cells/well and incubated for 24 h at 37 °C in 5% CO_2_. Cells were then exposed for 24 h to IAE (WP IAE: 0–2500 μg/mL or OP IAE: 0–400 μg/mL). Cell viability was quantified by MTT assay (Alfa Aesar, L11939). After exposure, the medium was removed and cells were rinsed once with phosphate-buffered saline (PBS; 137 mM NaCl, 2.7 mM KCl, 10 mM Na_2_HPO_4_, 1.8 mM KH_2_PO_4_). Fresh DMEM containing MTT (0.5 mg/mL) was added, and plates were incubated for 4 h at 37 °C. Formazan crystals were dissolved in 300 μL DMSO (Cyrus Bioscience, 101–67–68–5), and absorbance (A_570 nm_) was recorded on a SpectraMax i3 microplate reader (Molecular Devices). Results were analyzed using *t* tests.

### 2.5 Detection of Intracellular ROS Generation

To assess intracellular ROS generation, hydrogen peroxide (H_2_O_2_) was used as a representative marker of ROS levels following IAE treatment. SH-SY5Y cells with or without α-Syn overexpression were seeded in 96-well plates (α-Plus, 116196–3SB) at 3.5 × 10^4^ cells/well and incubated for 24 h at 37 °C. Cells were loaded with OxiVision Green H_2_O_2_ sensor (AAT Bioquest, 11503) for 1 h and then exposed to IAE for 80 min at 37 °C. The working concentrations were OP IAE: 55 μg/mL, WP IAE: 1000 μg/mL. Fluorescence was measured on a SpectraMax iD3 microplate reader (*E*_x_/*E*_m_: 490/525 nm). As a positive control, defined concentrations of H_2_O_2_ were also applied to validate assay performance.

### 2.6 Mitochondrial Membrane Potential Assay

Mitochondrial membrane potential was assessed using JC-1 (Abcam, ab113850) following the manufacturer’s instructions. Carbonyl cyanide *m*-chlorophenyl hydrazone (CCCP) disrupts mitochondrial function by collapsing the proton gradient across the inner mitochondrial membrane, thereby uncoupling electron transport from adenosine triphosphate synthesis and inducing mitochondrial depolarization. In this assay, carbonyl cyanide *m*-chlorophenyl hydrazone served as a positive control and was applied in accordance with the manufacturer’s recommendations. SH-SY5Y cells with or without expressing α-Syn were seeded in 96-well plates (α-Plus, 116196–3SB) at 3.5 × 10^4^ cells per well and incubated for 24 h at 37 °C (5% CO_2_). Cells were then exposed for 150 min at 37 °C to IAEs at the indicated concentrations (OP IAE: 55 μg/mL, WP IAE: 1000 μg/mL). Then, cells were incubated with 100 μL JC-1 working solution for 10 min, washed twice with 1× dilution buffer, and fluorescence was measured on a SpectraMax iD3 (Molecular Devices) at *E*_x_/*E*_m_ 475/550 nm for JC-1 monomers and *E*_x_/*E*_m_ 535/590 nm for aggregates. A Mitochondrial membrane potential index was calculated as the aggregate/monomer fluorescence ratio to indicate the mitochondria’s health/unhealth ratio.

### 2.7 ATP Assay

ATP levels were quantified using a luminescent ATP assay following the manufacturer’s instructions (Abcam, ab113849). In this assay, carbonyl cyanide *m*-chlorophenyl hydrazone served as a positive control and was applied in accordance with the manufacturer’s recommendations. SH-SY5Y cells, with or without α-Syn expression, were seeded in 96-well plates (α-Plus, 116,196–3SB) at 3.5 × 10^4^ cells per well and incubated for 24 h at 37 °C (5% CO_2_). Cells were then exposed for 150 min at 37 °C to IAE at the indicated concentrations (OP IAE: 55 μg/mL, WP IAE: 1000 μg/mL). After exposure, 50 μL of the supplied detergent/lysis reagent was added to each well and mixed for 5 min, followed by 50 μL of substrate solution with an additional 5 min mixing. Plates were incubated in the dark for 10 min at room temperature, and luminescence was recorded on a SpectraMax iD3 (Molecular Devices). Blank-subtracted relative light units were normalized to untreated controls, and statistical significance was evaluated using *t* tests.

### 2.8 Caspase-1/Caspase −3 Activity Detection

SH-SY5Y cells (1.0 × 10^6^ per well), with or without α-Syn overexpression, were exposed for 24 h at 37 °C to IAE at the indicated concentrations (OP IAE: 55 μg/mL, WP IAE: 1000 μg/mL). Cells were then lysed in 50 μL of manufacturer-supplied lysis buffer, and total protein was quantified by Bradford assay (Scientific Biotech Corp., BR01–500). Caspase-1 and caspase-3 activities were measured using colorimetric kits ab273268 (Abcam) and K106–25 (BioVision), respectively, with YVAD-pNA (caspase-1) and DEVD-pNA (caspase-3) substrates. Each reaction contained 150 μg total protein, 50 μL of 2× reaction buffer, and 5 μL of 4 mM substrate (final 200 μM), and was incubated for 2 h at 37 °C. Absorbance (A_400 nm_) was recorded on a microplate reader (SpectraMax i3, Molecular Devices). Caspase activity was expressed as the OD_400_ ratio relative to the appropriate control, and group differences were evaluated using *t* tests.

### 2.9 Autophagy Activity Detection

SH-SY5Y cells, with or without α-syn overexpression, were seeded at 3.5 × 10^4^ cells per well in 96-well plates (α-plus, 116196–3SB) and incubated for 24 h at 37 °C. Autophagy activity was assessed after exposure to IAE for 4 h at 37 °C using the following concentrations (OP IAE: 55 μg/mL, WP IAE: 1000 μg/mL). Cells were then incubated with the manufacturer’s autophagy detection reagent for 30 min, then washed with PBS, and added with 100 μL of 1× assay buffer (Abcam, ab139484). Fluorescence was measured at *E*_x_/*E*_m_ 475/550 nm using a microplate reader (SpectraMax iD3, Molecular Devices). Rapamycin induces autophagy by inhibiting mTOR complex 1, thereby activating the autophagy initiation machinery and promoting the degradation of proteins and organelles. Here, rapamycin was used as a positive control according to manufacturer’s instruction. Data was analyzed relative to matched controls, and group differences were evaluated using *t* tests.

### 2.10 Antioxidant Treatment

The MTT assay, intracellular ROS measurement, caspase-1/-3 activity assays, autophagy activity, and mitochondrial membrane potential measurements were performed as described above, using either IAE or CAE at OP 55 μg/mL or WP 1000 μg/mL, with or without cotreatment by rutin (0.2 mM; Acros Organics) or *N*-acetylcysteine (NAC, 0.5 mM; Acros Organics). Antioxidant concentrations were determined by MTT assay and selected to ensure that cell viability remained above 95% following antioxidant treatment alone (Supporting Information Figure S2). Statistical comparisons were made using *t* tests.

### 2.11 Fluorescence Correlation Spectroscopy (FCS) Experiments

FCS measurements were performed on a custom confocal setup built on a Nikon Ti Eclipse microscope. Excitation was provided by 470 nm (PicoQuant, LDH–P–C-470M) and 560 nm (PicoQuant, LDH-D-TA-560B) diode lasers, combined by a quad-band dichroic (Semrock, Di01-R405/488/561/635) and focused into the sample using a 100× Nikon Apochromat oil-immersion objective (NA 1.40). Emitted fluorescence passed through a quad-notch filter (Semrock, NF03–405/488/561/635), a secondary dichroic (Chroma, T585lpxr), and band-pass filters (Chroma HQ520/30 for eGFP; Chroma 645FA for mApple), and was detected by single-photon avalanche photodiodes (Micro Photon Devices, MPD-5C5T). Intensity traces (60s) were acquired with SymPhoTime (PicoQuant), and autocorrelation functions were fitted to either a one-component 3D free-diffusion model (eq 1) or an anomalous-diffusion model (eq 2):

1 $$G\left(\tau \right)=\left(1+\frac{T_{R}e^{-\tau /\tau_{R}}}{1-T_{R}}\right)\left[\frac{1}{\langle C\rangle V_{eff}}\times \frac{1}{{\left(1+\frac{\tau }{\tau_{D}}\right)\left(1+\frac{\tau }{\omega^{2}\tau_{D}}\right)}^{0.5}}\right]$$

2 $$G\left(\tau \right)=\left(1+\frac{T_{R}e^{-\tau /\tau_{R}}}{1-T_{R}}\right)\left\{\frac{1}{\langle C\rangle V_{eff}}\times \frac{1}{{\left[1+{\left(\frac{\tau }{\tau_{D}}\right)}^{\alpha }\right]\left[1+\frac{\tau^{\alpha }}{\omega^{2}{\tau_{D}}^{\alpha }}\right]}^{0.5}}\right\}$$

In these equations, *V*_eff_ refers to the effective excitation volume, with an axial (*z*_0_) to lateral (*r*_0_) dimension ratio denoted by *w* (=*z*_0_/*r*_0_), and < *C*> is the average concentration of observed molecules. *T*_R_ and τ_R_ refer to the triplet state population and its triplet state relaxation time, respectively, while τ is the correlation time, and τ_D_ represents the diffusion time. Calibration of *w* and *V*_eff_ awas achieved using a standard dye, R6G (*D* = 414 μm^2^/s)^44^ and R640 (*D* = 470 μm^2^/s). The anomalous factor α accounts for the effects of the intracellular environment heterogeneity on diffusion.^45–47^

### 2.12 Statistical Analysis

Data are presented as mean ± standard deviation (SD) from at least three independent experiments. All statistical analyses were performed using OriginPro 2023b (OriginLab Corporation, Northampton, MA, USA). To evaluate the main effects and interactions among multiple variables (e.g., cell type, incense type, and aerosol fraction), data were analyzed using two-way or three-way analysis of variance (ANOVA), as appropriate. When significant effects were detected by ANOVA, Tukey’s post hoc test was applied for multiple comparisons. For direct comparisons between two specific treatment conditions, data from at least three independent experiments are presented as mean ± SD, and unpaired *t* tests were used for statistical analysis. Statistical significance was defined as ns (*p* ≥ 0.10), trend (#, 0.05 < 0.10), *p < 0.05, **p < 0.01, and ***p < 0.001. All statistical tests were two-tailed.

## 3 Results

### 3.1 α-Syn Overexpression Exacerbates IAE-Induced Cell Death, ROS Generation, and Mitochondrial Dysfunction in SH-SY5Y Cells

Previous studies have demonstrated that IAEs decrease cell viability, elevate intracellular ROS, and disrupt mitochondrial function in human cell lines, with SH-SY5Y neuroblastoma cells exhibiting greater sensitivity than A549 or HEK293T cells.^26^ Given that α-Syn misfolding and aggregation are pathological hallmarks of PD.^48–51^ Previously, we utilized SH-SY5Y cells overexpressing α-Syn, a validated cellular model for assessing CAE impact.^38,39,52–55^ Here, we investigated whether α-Syn overexpression aggravates IAE-induced cytotoxicity.

Cells were treated with size-fractionated IAEs (Fractions I–IV) from three incense types, and viability was assessed after 24 h. Quantitative analysis from three independent experiments (*n* = 3) revealed that α-Syn overexpression markedly exacerbated susceptibility to IAEs (Figure 1 and Supporting Information Figure S3). Across all incense types and size fractions, α-Syn overexpressing cells exhibited consistently lower IC_50_ values compared to nonexpressing controls (Table 1). Among the tested incense types, type A IAEs elicited the strongest cytotoxicity, yielding the lowest IC_50_ values in both OP and WP extracts treatments compared to types B and C. A distinct size-dependent toxicity was also observed; extracts derived from smaller aerodynamic particles (Fractions III and IV) generally induced greater toxicity than those from coarser particles (Fraction I), consistent with our previous report.^26^ Moreover, Fraction III constitutes the most abundant aerosol population by mass. Based on these toxicity profiles—and previous data indicating that type A induces the highest ROS accumulation and caspase activation.^26^ Therefore, type A OP-IAE and WP-IAE derived from Fraction III were selected for subsequent experiments to evaluate the synergistic effects of IAE and α-Syn.

**Figure 1:**
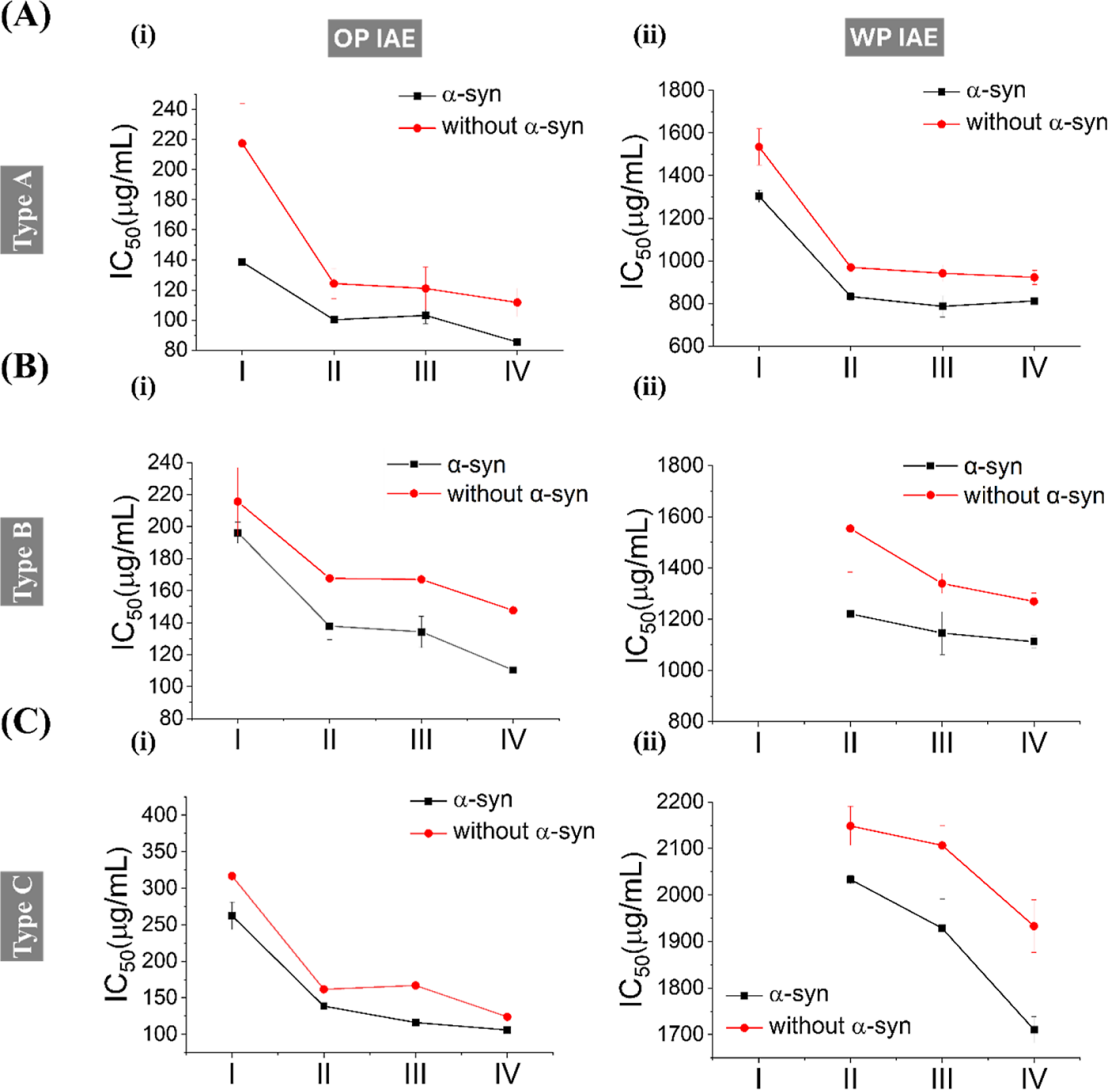
IC_50_ values of incense aerosol extracts (IAEs) derived from three incense types—(A) type A (sandalwood-dominant), (B) type B (agarwood-dominant), and (C) type C (binchotan charcoal-dominant)—in SH-SY5Y cells with or without α-Syn overexpression. IC_50_ values were determined for (i) OP IAE and (ii) WP IAE extracts across particle size fractions I–IV (see Experimental Section). Data represent the mean values from three independent experiments.

**Table 1:** IC_50_ Values of OP IAE Extract and WP IAE Extracts. I–VI Indicate the Size of Incense Aerosol Obtained with MOUDI and the Size Range is Listed in the Table

		IC50 (μg/mL)			
		SH-SY5Y			
		no α-synuclein overexpression^a^		with α-synuclein overexpression	
		OP	WP	OP	WP
**I (>0.56 μm)**	type A	217.5 ± 26.4	1534.8 ± 85.6	138.6 ± 6.0	1302.9 ± 29.4
	type B	215.6 ± 21.4	N.D	196.2 ± 6.7	N.D
	type C	316.8 ± 2.0	N.D	262.3 ± 18.6	N.D
**II (0.56–0.18 μm)**	type A	124.3 ± 10.0	969.6 ± 13.1	100.2 ± 5.6	832.8 ± 18.4
	type B	167.7 ± 1.3	1554.0 ± 170.0	137.8 ± 8.5	1221.1 ± 53.9
	type C	161.7 ± 1.8	2148.5 ± 42.0	138.6 ± 1.9	2033.4 ± 8.4
**III (0.18–0.1 μm)**	type A	121.0 ± 14.2	942.1 ± 39.2	103.1 ± 5.4	786.9 ± 50.4
	type B	166.9 ± 0.5	1339.7 ± 71.8	134.2 ± 9.7	1146.0 ± 84.0
	type C	138.0 ± 1.2	2106.7 ± 43.0	116.23 ± 2.5	1928.6 ± 63.2
**IV (<0.1 μm)**	type A	111.7 ± 9.4	923.0 ± 33.4	85.4 ± 3.1	812.8 ± 11.0
	type B	147.6 ± 4.4	1269.3 ± 6.8	110.3 ± 18.3	1112.0 ± 23.9
	type C	124.0 ± 3.8	1933.1 ± 56.6	106.23 ± 1.7	1710.6 ± 28.2

a Indicate data from the reference.

To determine whether α-Syn overexpression amplifies IAE-induced oxidative stress, SH-SY5Y cells were transfected with α-Syn and exposed to IAE simultaneously. While α-Syn overexpression and IAE exposure independently elevated intracellular H_2_O_2_, their combination produced a synergistic increase in ROS levels. This effect was consistent across both OP-IAE and WP-IAE treatments (Figure 2A).

**Figure 2:**
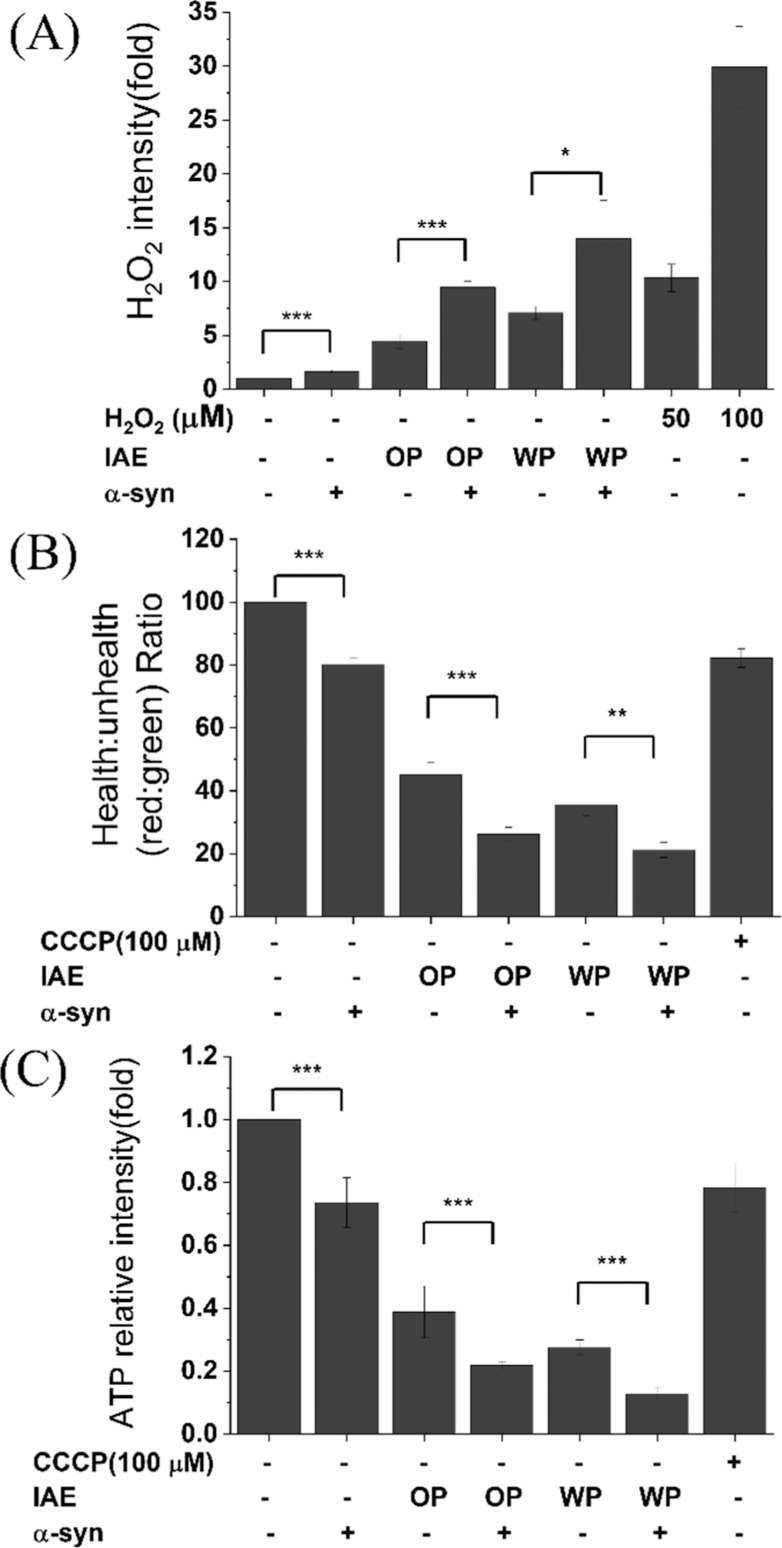
Effects of type A IAEs on cellular stress response in SH-SY5Y cells with or without α-Syn overexpression. Shown are (A) intracellular H_2_O_2_ generation, (B) mitochondrial membrane potential, and (C) intracellular ATP levels following treatment with OP IAE or WP IAE. Data are expressed related to untreated controls. Each condition conducted three repeated experiments. *, **, and *** indicate significance (*p < 0.05, **p < 0.01, ***p < 0.001).

We next assessed mitochondrial dysfunction, a key driver of PD pathology often linked to impaired Complex I activity and mtDNA damage.^56–58^ Following 24 h of α-Syn overexpression and 150 min of IAE treatment, cells overexpressing α-Syn exhibited a significantly more pronounced decline in mitochondrial membrane potential (MMP) compared to controls (Figure 2B). As mitochondria are central to bioenergetics,^59–61^ cellular ATP levels were subsequently measured. Consistently, α-Syn -overexpressing cells treated with either OP-IAE or WP-IAE displayed significantly lower ATP production than controls (Figure 2C). Collectively, these results demonstrate a synergistic decline in mitochondrial function (Figure 2B,C), suggesting that α-Syn overexpression sensitizes neuronal cells to IAE-induced oxidative and mitochondrial stress, leading to enhanced bioenergetic failure.

### 3.2 α-Syn Overexpression Exacerbates IAE-Induced Apoptosis and Pyroptosis but Attenuates IAE-Induced Autophagy Activity in SH-SY5Y Cells

Incense aerosols are recognized inducers of programmed cell death (PCD)—including apoptosis, pyroptosis, and autophagy—mediated largely through ROS generation, inflammasome activation, and mitochondrial injury.^62–70^ Building on our prior observations that α-Syn aggravates CAE-induced cytotoxicity accompanying with aggravating cell death^38^ ^,^ ^39^ we investigated the specific engagement of these pathways in SH-SY5Y cells exposed to IAEs.

As presented in Figure 3A,B, exposure to both OP-IAE and WP-IAE resulted in significant elevations in caspase-3 and caspase-1 activities, canonical markers of apoptosis and pyroptosis, respectively. Notably, these enzymatic activities were markedly higher in α-Syn -overexpressing cells compared to controls. This data confirms that α-Syn overexpression sensitizes neuronal cells to IAE-induced stress, shifting the cellular response toward apoptotic and pyroptotic cell death execution.

**Figure 3:**
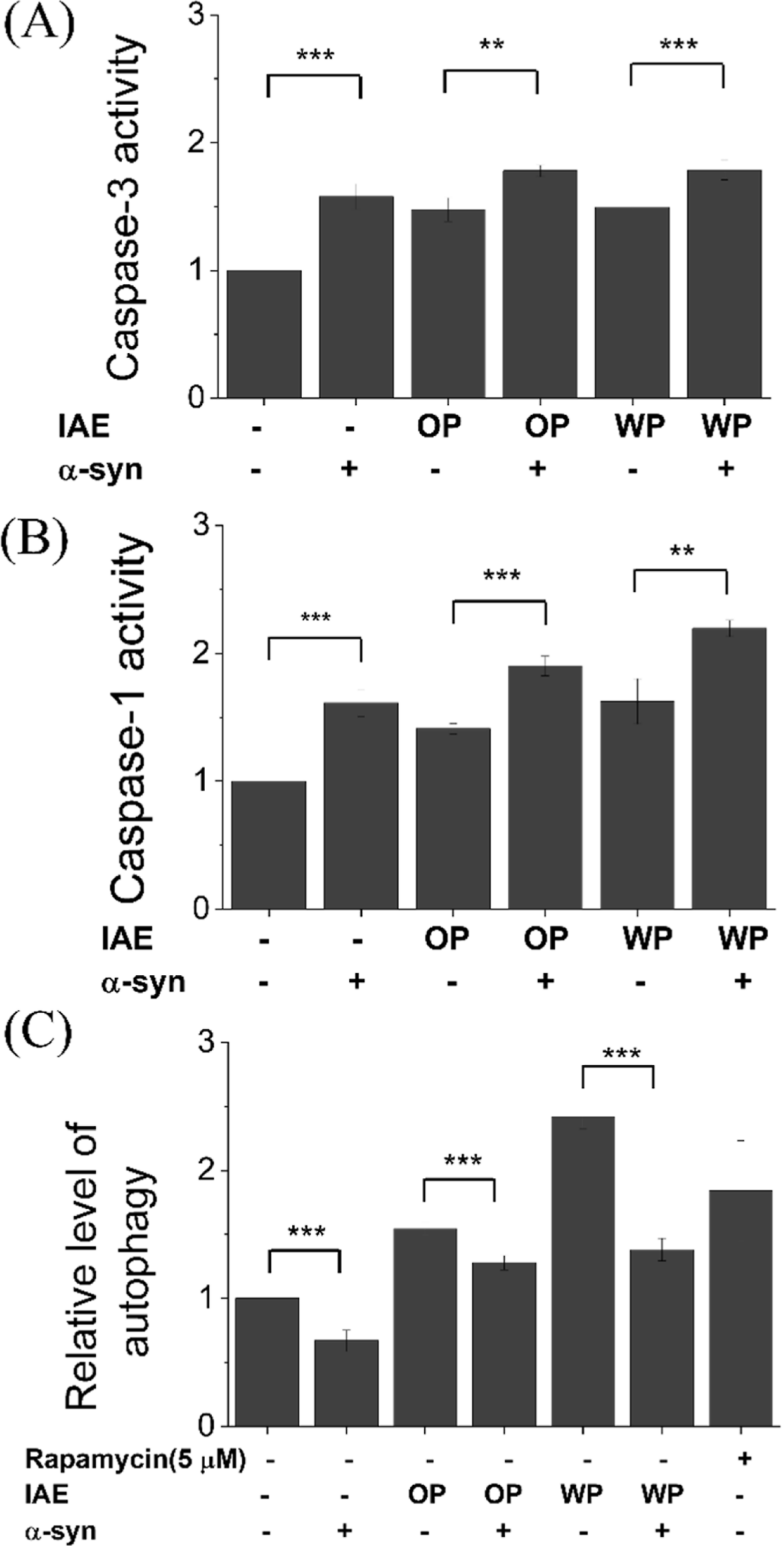
Effects of type A IAE on PCD and autophagic response in SH-SY5Y cells with or without α-Syn overexpression. (A) Caspase-1 activity (pyroptosis), (B) caspase-3 activity (apoptosis), and (C) autophagy activity were quantified following treatment with OP IAE or WP IAE. Data represent mean ± SD from three repeated experiments; *, *, and *** indicate *p* < 0.05, *p* < 0.01, and *p* < 0.001, respectively.

In contrast, autophagy activity exhibited a divergent pattern (Figure 3C). Although IAE treatment alone enhanced autophagy—likely as a compensatory stress response—α-Syn overexpression markedly reduced autophagy levels, both in untreated cells and in those coexposed to IAEs. This suppression suggests that α-Syn disrupts autophagic flux, diminishing the cell’s ability to manage proteotoxic and oxidative burdens.

Therefore, these results demonstrate that α-Syn overexpression exerts a dual deleterious effect: it synergistically amplifies IAE-induced apoptosis and pyroptosis while simultaneously blunting the protective autophagic response. This mechanism likely contributes to the enhanced neuronal vulnerability observed in PD pathogenesis.^71–73^

### 3.3 IAE Treatment Alters the Oligomeric State of α-Syn and Promotes the Dissociation of Oligomeric α-Syn into Monomeric Form

Previous studies have established that CAEs disrupt α-syn homeostasis, shifting oligomeric species toward monomers and driving puncta formation colocalized with lysosomes, thereby implicating aerosol pollutants in synucleinopathy progression.^39,43^ To examine whether IAEs exert similar effects, IAE derived from the most abundant population (Fraction III) was selected to investigate its influence. We assessed the oligomeric state of α-Syn in SH-SY5Y cells coexpressing eGFP−α-Syn and mApple−α-Syn following OP-IAE or WP-IAE treatment. In untreated control cells, α-Syn exhibited a homogeneous distribution consistent with a stable oligomeric equilibrium (Figures 4A­(i) and 5A­(i)). Distinct from CAE-treated cells, which display robust puncta formation,^39,43^ IAE exposure did not trigger visible intracellular α-Syn puncta or aggregates at either 24 or 48 h (Figures 4A­(ii–iii), 5A­(ii–iii); Supporting Information Figures S4 and S5).

**Figure 4:**
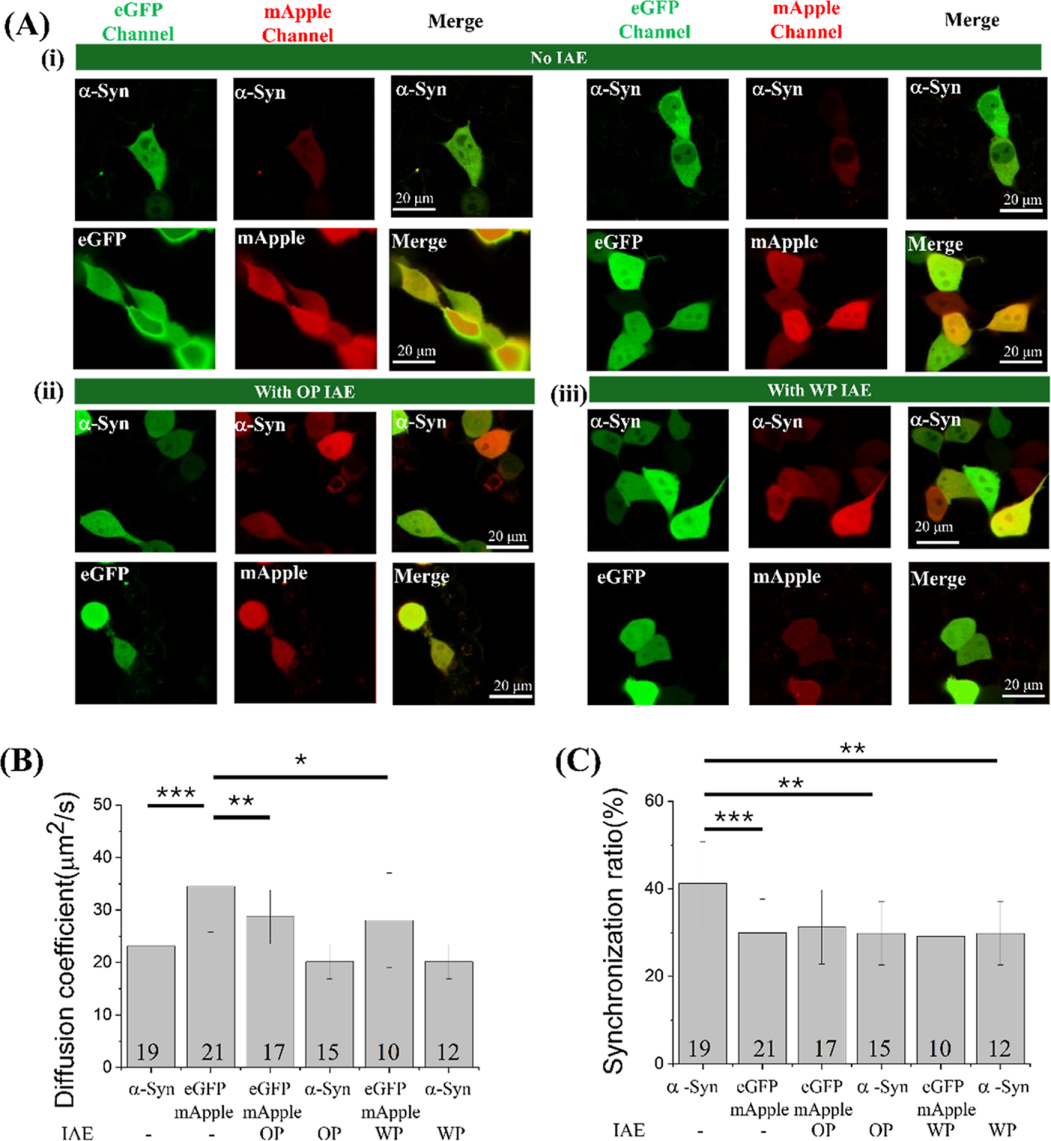
(A) Confocal imaging and fluorescence correlation analysis of SH-SY5Y cells coexpressing eGFP−α-Syn and mApple−α-Syn following 24 h treatment with IAE. Representative confocal images of (i) untreated cells and (ii) cells treated with OP IAE or (iii) WP IAE from type A (sandalwood-dominant) are shown. Scale bar = 10 μm. (B) Cross-correlation ratios and (C) diffusion coefficients derived from FCS and FCCS in the same cells. Each FCS curve was fitted with a one-component 3D diffusion model. Data represent mean ± SD. Numbers indicate the total number of cells analyzed. *, **, *** indicate *p* < 0.05, 0.01, and 0.001, respectively.

Fluorescence correlation spectroscopy (FCS) and fluorescence cross-correlation spectroscopy (FCCS) provided quantitative insight into the diffusion dynamics and oligomeric state. For untreated cells transfected with eGFP and mApple vectors, diffusion coefficients and concentrations were consistent with established literature values (Figure 4B, Tables 2 and 3).^39,43^ Following OP-IAE exposure, the diffusion coefficient of eGFP decreased slightly (e.g., 28.73 ± 5.12 μm^2^/s), while the anomalous diffusion exponent (α) dropped to 0.73–0.76 (compared to 0.95 ± 0.10 in controls). This reduction suggests increased intracellular crowding or hindrance, a phenomenon previously reported under stress conditions.^46,74–80^ Importantly, the cross-correlation ratio for eGFP/mApple controls remained at the baseline level of ∼30%, confirming a monomeric state (Figure 4B,C and Table 2). Comparable effects on cellular environment were observed with WP-IAE treatment (Table 3).

**Table 2:** Diffusion Coefficients, Concentrations, and Anomalous Parameters of eGFP, mApple, eGFP−α-Syn, and mApple−α-Syn in SH-SY5Y Cells after 24 h or 48 h of Transfection with OP IAE (0.18 μm–0.10 μm)

	no IAE				with IAE			
	eGFP and mApple		eGFP−α-Syn and mApple−α-Syn		eGFP and mApple		eGFP−α-Syn and mApple−α-Syn	
	24 h (*N* = 7, *n* = 21)	48 h (*N* = 3, *n* = 20)	24 h (*N* = 6, *n* = 19)	48 h (*N* = 6, *n* = 17)	24 h (*N* = 5, *n* = 17)	48 h (*N* = 4, *n* = 14)	24 h (*N* = 5, *n* = 15)	48 h (*N* = 4, *N* = 17)
diffusion coefficient (μm^2^/s) (free 3D diffusion fitting)	34.5 ± 8.7	35.2 ± 6.6	23.1 ± 8.0	19.2 ± 8.4	28.7 ± 5.1	23.9 ± 8.7	20.1 ± 3.3	19.7 ± 6.3
eGFP concentration (nM)	269.9 ± 192.8	288.9 ± 261.7	280.8 ± 308.4	473.1 ± 335.0	295.6 ± 58.8	276.2 ± 152.7	243.4 ± 72.7	338.9 ± 218.2
mApple concentration (nM)	245.9 ± 145.1	261.4 ± 129.3	303.5 ± 418.6	262.7 ± 262.6	241.1 ± 3.4	261.8 ± 76.5	232.9 ± 74.4	294.1 ± 118.8
transport coefficient (μm^2^/s) (anomalous fitting)	41.0 ± 6.8 (α = 0.95 ± 0.10)	39.9 ± 15.2 (α = 0.78 ± 0.26)	30.8 ± 4.4 (α = 0.82 ± 0.16)	31.4 ± 5.4 (α = 0.77 ± 0.06	37.1 ± 5.1 (α = 0.76 ± 0.06)	34.9 ± 7.4 (α = 0.73 ± 0.12)	37.9 ± 5.1 (α = 0.77 ± 0.13)	36.7 ± 3.9 (α = 0.73 ± 0.06)
FCCS ratio (%)	29.9 ± 7.8	29.1 ± 5.7	41.2 ± 5.6	60.6 ± 9.9	29.28.3	30.2 ± 9.3	29.8 ± 7.2	33.8 ± 8.6

**Table 3:** Diffusion Coefficients, Concentrations, and Anomalous Parameters of eGFP, mApple, eGFP−α-Syn, and mApple−α-Syn in SH-SY5Y Cells after 24 or 48 h of Transfection with WP IAE (0.18 μm–0.10 μm)

	no IAE				with IAE			
	eGFP and mApple		eGFP−α-Syn and mApple−α-Syn		eGFP and mApple		eGFP−α-Syn and mApple−α-Syn	
	24 h (*N* = 7, *n* = 21)	48 h (*N* = 3, *n* = 20)	24 h (*N* = 6, *n* = 19)	48 h (*N* = 6, *n* = 17)	24 h (*N* = 3, *n* = 10)	48 h (*N* = 3, *n* = 12)	24 h (*N* = 4, *n* = 12)	48 h (*N* = 3, *n* = 15)
diffusion coefficient (μm^2^/s) (free 3D diffusion fitting)	34.5 ± 8.7	35.2 ± 6.6	23.1 ± 8.0	19.2 ± 8.4	28.1 ± 9.0	23.7 ± 12.7	20.9 ± 6.0	22.5 ± 12.3
eGFP concentration (nM)	269.9 ± 192.8	288.9 ± 261.7	280.8 ± 308.4	473.1 ± 335.0	332.3 ± 88.4	303.7 ± 164.5	325.1 ± 140.8	282.8 ± 190.8
mApple concentration (nM)	214.9 ± 245.1	261.4 ± 129.3	203.5 ± 418.6	262.7 ± 262.6	320.5 ± 60.2	227.8 ± 27.6	287.5 ± 234.9	230.6 ± 118.8
transport coefficient (μm^2^/s) (anomalous fitting)	41.0 ± 6.8 (α = 0.95 ± 0.10)	39.9 ± 15.2 (α = 0.78 ± 0.26)	30.8 ± 4.4 (α = 0.82 ± 0.16)	31.4 ± 5.4 (α = 0.77 ± 0.06)	36.3 ± 7.6 (α = 0.75 ± 0.14)	34.9 ± 7.4 (α = 0.74 ± 0.63)	35.7 ± 6.3 (α = 0.77 ± 0.11)	43.9 ± 12.6 (α = 0.73 ± 0.14)
FCCS ratio (%)	29.9 ± 7.8	29.1 ± 5.7	41.2 ± 9.6	60.6 ± 9.9	7.5 ± 9.5	27.8 ± 11.3	28.8 ± 8.0	21.1 ± 9.9

We next analyzed the oligomeric state of eGFP−α-Syn. In vehicle-treated cells, eGFP−α-Syn maintained an oligomeric state, characterized by a diffusion coefficient of 23.1 ± 8.0 μm^2^/s and a cross-correlation ratio of 41.2 ± 5.6%, consistent with our prior report.^39,43^ Analysis of eGFP−α-Syn revealed that OP-IAE treatment did not significantly alter diffusion coefficients compared to untreated cells (Figure 4B and Table 2). However, anomalous factors of 0.73–0.79 and reduced cross-correlation ratios (∼30%) indicate a monomeric state (Figure 4C and Table 2). Comparable results were observed for WP-IAE, further supporting that both OP-IAE and WP-IAE promote a shift toward the monomeric form of α-Syn in SH-SY5Y cells (Figure 4C and Table 3).

Importantly, this monomeric shift persisted at 48 h. Diffusion coefficients, anomalous exponents, and cross-correlation ratios remained stable and comparable to the 24 h time point (Figure 5B,C, Tables 2 and 3). The absence of puncta even at later time points suggests that IAEs do not merely delay α-Syn oligomerization or puncta formation; rather, they maintain α-Syn in a sustained monomeric state. This persistent monomerization distinguishes IAE exposure from CAE exposure, in which a progressive shift toward puncta formation and lysosomal colocalization is observed.^39^ Therefore, our results imply that exposure to IAEs leads to a sustained disruption of α-Syn oligomerization equilibrium, favoring the maintenance of the monomeric form in the cytosol over time.

**Figure 5:**
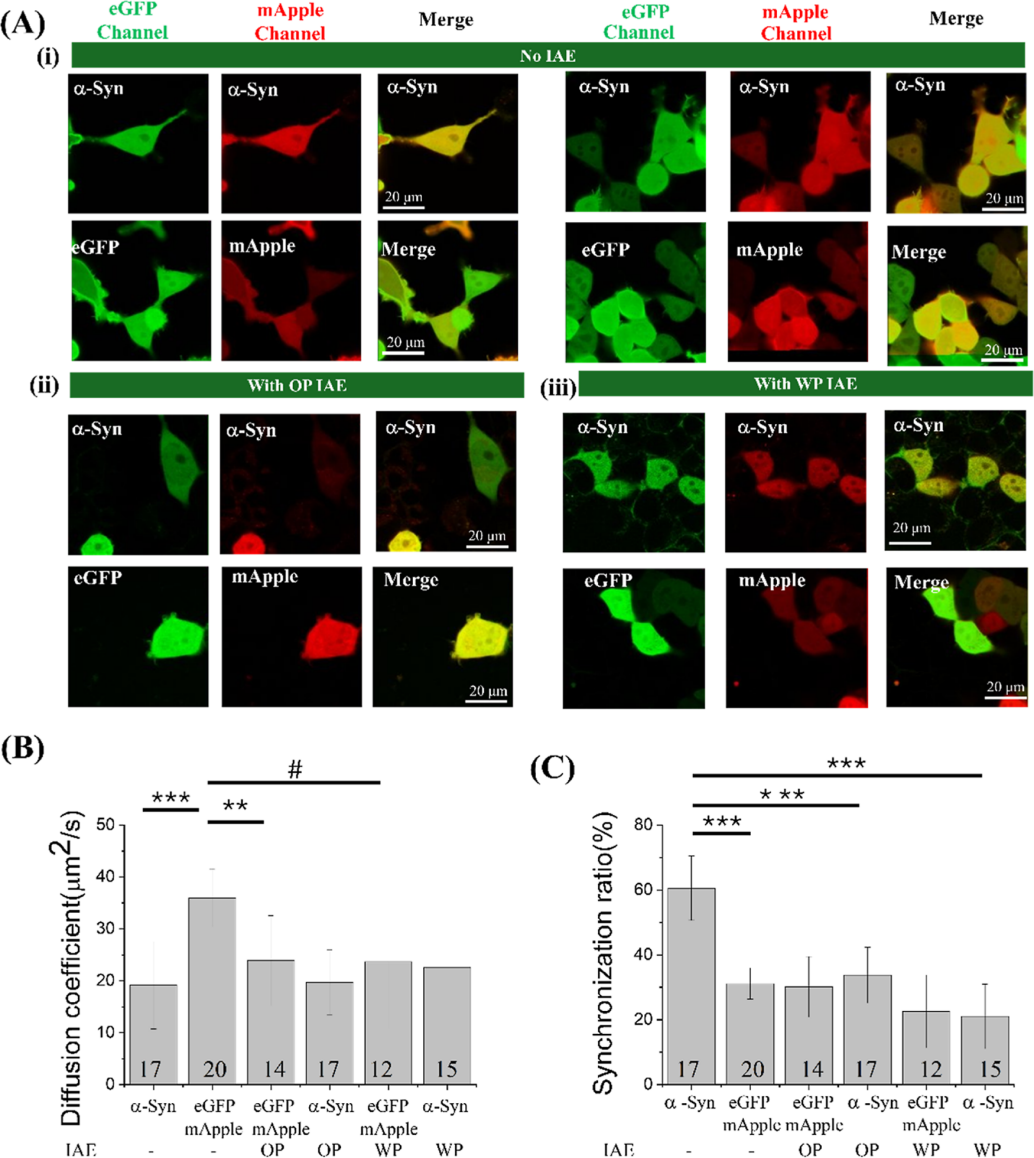
(A) Confocal imaging and FCS analysis of SH-SY5Y cells coexpressing eGFP−α-Syn and mApple−α-Syn following 48 h treatment with IAE. Representative confocal images of (i) untreated cells and (ii) cells treated with OP IAE or (iii) WP IAE from type A (sandalwood-dominant) are shown. Scale bar = 10 μm. (B) Cross-correlation ratios and (C) diffusion coefficients derived from FCS) and FCCS in the same cells. Each FCS curve was fitted with a one-component 3D diffusion model. Data represent mean ± SD; numbers indicate the total number of cells analyzed. #,0.05 ≤ *p* < 0.10;*p < 0.05;**p 0.01;***p < 0.001, respectively.

### 3.4 Differential Rescue Efficacy: OP-IAE-Induced Toxicity is Resistant to Antioxidant Intervention Despite Rescue in CAE and WP-IAE Models

Rutin, a plant-derived flavonoid glycoside with broad antioxidant and neuroprotective activities,^81–85^ was previously shown to rescue combustion CAEs–induced cytotoxicity in several cell lines (A549, HEK293T, and SH-SY5Y).^38^ However, its protective effect against CAEs has not been evaluated in SH-SY5Y neuroblastoma cells overexpressing α-syn. Similarity, NAC, a cysteine prodrug and glutathione precursor, well-known for its potent antioxidant and anti-inflammatory effects in models of neurological injury and oxidative stress,^86–88^ was selected to compare rescue potentials. To evaluate the therapeutic potential of antioxidants, we selected Rutin (0.2 mM) and NAC (0.5 mM) based on dose-optimization assays that confirmed these concentrations as the maximal nontoxic doses (>95% viability) for this specific cellular model (Supporting Information Figure S2). Consequently, these concentrations were utilized to compare their rescue potential against the most potent incense type (type A) relative to CAE.

We first measured intracellular H_2_O_2_ levels to assess the effect of antioxidant cotreatment on ROS accumulation (Figure 6). Both IAE and CAE exposure caused a significant increase in intracellular H_2_O_2_, with a more pronounced rise observed in α-Syn–overexpressing cells. Antioxidant cotreatment significantly alleviated this ROS elevation, with NAC demonstrating superior scavenging efficacy compared to Rutin. While Rutin treatment provided a statistically significant reduction (*p* < 0.05), it failed to fully counteract the oxidative surge in α-Syn cells. In contrast, NAC treatment resulted in a profound suppression of ROS (*p* < 0.001). Notably, the rescue efficacy differed between aerosol sources. In the OP-CAE treatment, NAC effectively neutralized the α-Syn-mediated ROS amplification, restoring levels to near-baseline. Conversely, despite the potent effects of NAC, α-Syn–overexpressing cells exposed to IAE maintained significantly higher residual ROS levels compared to their CAE-treated counterparts (*p* < 0.01).

**Figure 6:**
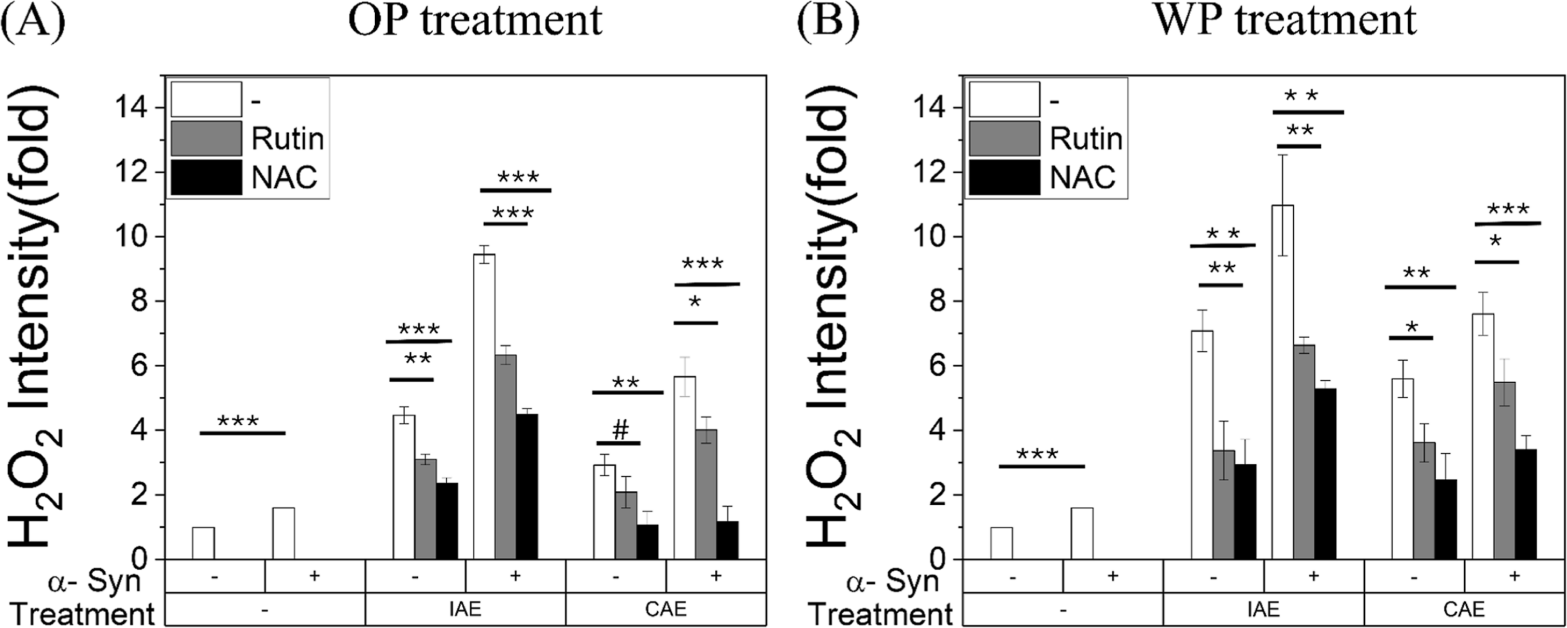
Intracellular ROS production in SH-SY5Y cells with or without α-Syn overexpression following 24 h treatment with (A) OP extracts treatment or (B) WP extracts treatment in the presence or absence of cotreatment with rutin (0.2 mM) or (ii) NAC (0.5 mM). ROS levels are expressed as a percentage of untreated controls (mean ± SD, *n* = 3). ns, *p* ≥ 0.10; #, 0.05 ≤ *p* < 0.10; *p < 0.05; **p < 0.01; ***p < 0.001 versus the corresponding IAE- or CAE-only condition.

Next, we determined whether the antioxidant-mediated reduction in ROS translated to improved cell survival by assessing shifts in IC_50_ values (Figure 7, Supporting Information Figure S6 and Table 4). Consistent with the oxidative stress data, antioxidant intervention significantly ameliorated cytotoxicity in the majority of treatment conditions (WP-IAE, WP-CAE, and OP-CAE); however, the magnitude of this rescue was dependent on the aerosol extract type. In CAE-treated groups, both Rutin and NAC induced significant elevations in IC_50_ values compared to untreated controls (*p* < 0.05 to *p* < 0.01). This protective effect was most pronounced in the OP-CAE condition, where NAC treatment in α-Syn–overexpressing cells increased the IC_50_ from 113.8 ± 4.6 to 280.7 ± 26.8 μg/mL (*p* < 0.01). In contrast, OP-IAE toxicity exhibited resistance to antioxidant rescue. Regardless of the antioxidant used or the presence of α-Syn, neither Rutin nor NAC produced a statistically significant improvement in cell survival (*p* > 0.05). Furthermore, the superior efficacy of NAC over Rutin was statistically evident in the context of α-Syn overexpression. While Rutin failed to provide significant rescue in WP-IAE and WP-CAE conditions (*p* > 0.05), NAC consistently maintained a significant protective effect (*p* < 0.05 to *p* < 0.01) across these same groups.

**Figure 7:**
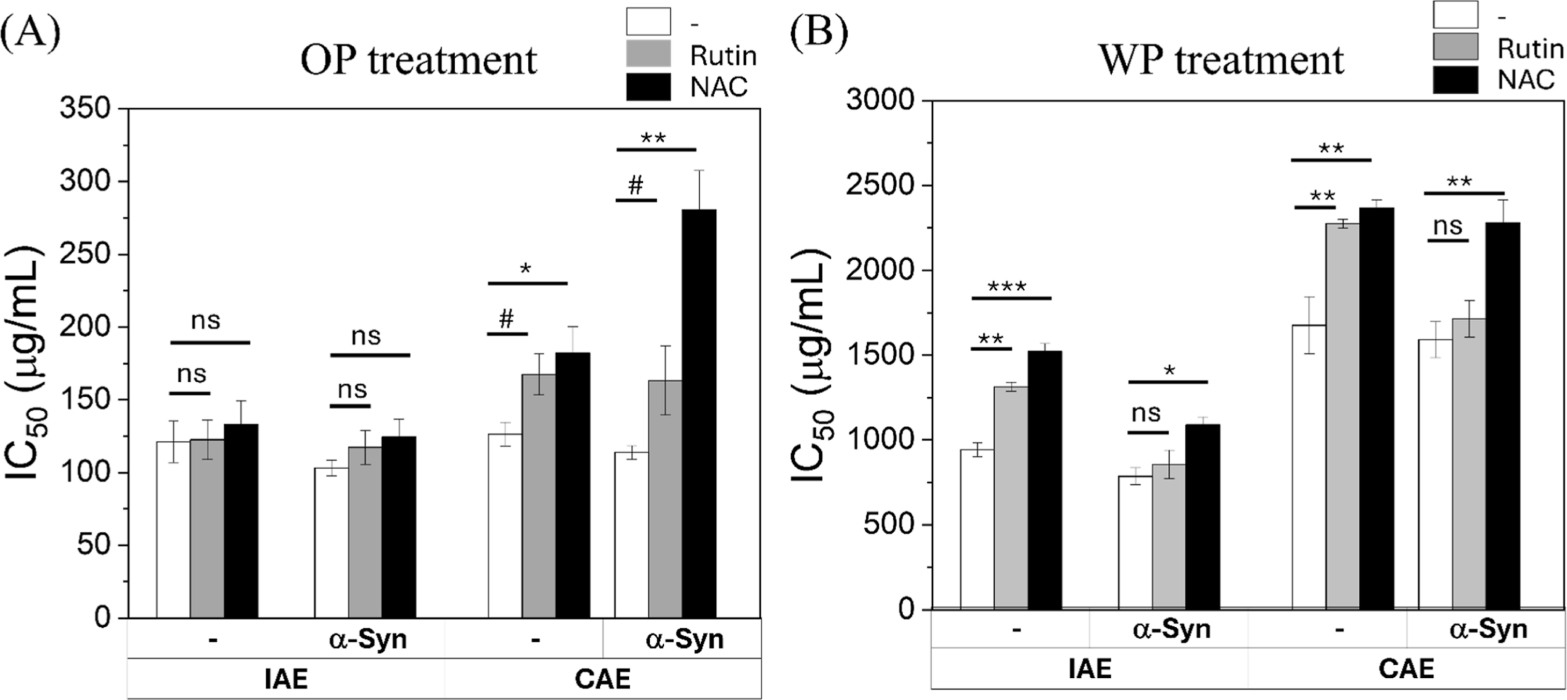
IC_50_ in SH-SY5Y cells with or without α-syn overexpression, and the protective effects of rutin and NAC following 24 h treatment with (A) OP/WP IAE derived from aerosol with diameters ranging from 0.18 μm–0.10 μm (type A incense) or (B) OP/WP CAE derived from aerosol with diameters ranging from 0.56 μm–0.32 μm, in the presence or absence of cotreatment with (i) rutin (0.2 mM) or (ii) *N*-acetylcysteine (NAC, 0.5 mM). Cell viability was assessed using the MTT assay and is presented as a percentage of untreated controls (mean ± SD, *n* = 3). Data are presented as mean ± SD from three independent experiments. Statistical significance was determined using paired *t* tests with a conservative approximation. ns, *p* ≥ 0.10; #, 0.05 ≤ *p* < 0.10; *p < 0.05; **p < 0.01; ***p < 0.001.

**Table 4:** IC_50_ Values of OP IAE (0.18 μm–0.10 μm, Most Abundant Fraction) and OP CAE (0.56 μm–0.32 μm, Most Abundant Fraction) with or without Antioxidative Reagents (Rutin and NAC)

		IC_50_ (μg/mL) in SH-SY5Y Cells					
		no α-synuclein overexpression			with α-synuclein overexpression		
		–	Rutin	NAC	–	Rutin	NAC
incense aerosol extract	OP	121.0 ± 14.2	122.4 ± 13.6	133.1 ± 16.3	103.1 ± 5.4	117.2 ± 11.7	124.3 ± 12.3
	WP	942.1 ± 39.2	1313.8 ± 26.7	1521.5 ± 46.3	786.9 ± 50.4	856.0 ± 8.3	1088.3 ± 4.7
cigarette aerosol extract	OP	121.0 ± 14.2	147.1 ± 34.1	182.02 ± 19.8	113.8 ± 4.6	141.9 ± 28.6	280.7 ± 26.8
	WP	1676.4 ± 167.8	2300.0 ± 21.8	2368.9 ± 44.3	1591.3 ± 106.1	1714.4 ± 273.8	2280.7 ± 133.6

### 3.5 Antioxidants Differentially Modulate PCD Pathways in OP-CAE vs OP-IAE Treated Cells but Fail to Restore the Native α-Syn Oligomeric State

The rationale for restricting downstream mechanistic profiling to the OP fractions was 2-fold, based on bioactivity and antioxidant responsiveness. First, the WP fractions (WP-IAE and WP-CAE) exhibited significantly higher IC_50_ values compared to their OP counterparts, indicating a lower baseline cytotoxic potency. Second, and more critically, viability assays revealed a fundamental divergence in rescue patterns: while the cytotoxicity of WP-IAE, WP-CAE, and OP-CAE was effectively reversed by antioxidant supplementation (Rutin or NAC), OP-IAE toxicity remained refractory to these rescue strategies. This suggests that WP fractions and OP-CAE share a common, ROS-dependent toxicity profile, whereas OP-IAE activates unique, ROS-independent death pathways.

To elucidate these distinct mechanisms, we focused exclusively on OP-IAE and OP-CAE to examine MMP, Caspase-1/3 activation, and autophagy. Quantitative analysis of three independent replicates using *t* tests confirmed the unique nature of OP-IAE toxicity. Consistent with the cell viability data, neither Rutin nor NAC alleviated the influence of OP-IAE treatment; statistical comparisons showed no significant reduction (*p* > 0.05) in Caspase-1 (Figure 8A) or Caspase-3 (Figure 8B) activation, nor any significant recovery of autophagy dysregulation (Figure 8C) or MMP levels (Figure 8D). In contrast, both antioxidants demonstrated robust rescue effects in OP-CAE treated cells. Unpaired *t*-test analysis confirmed that cotreatment significantly attenuated Caspase-1 (Figure 8A) and Caspase-3 (Figure 8B) activities (*p* < 0.05), restored autophagy to baseline levels (Figure 8C), and facilitated the significant recovery of MMP (Figure 8D). These data reinforce that OP-IAE toxicity engages persistent cellular injury mechanisms that are distinct from the scavenge-able oxidative stress observed in the other fractions.

**Figure 8:**
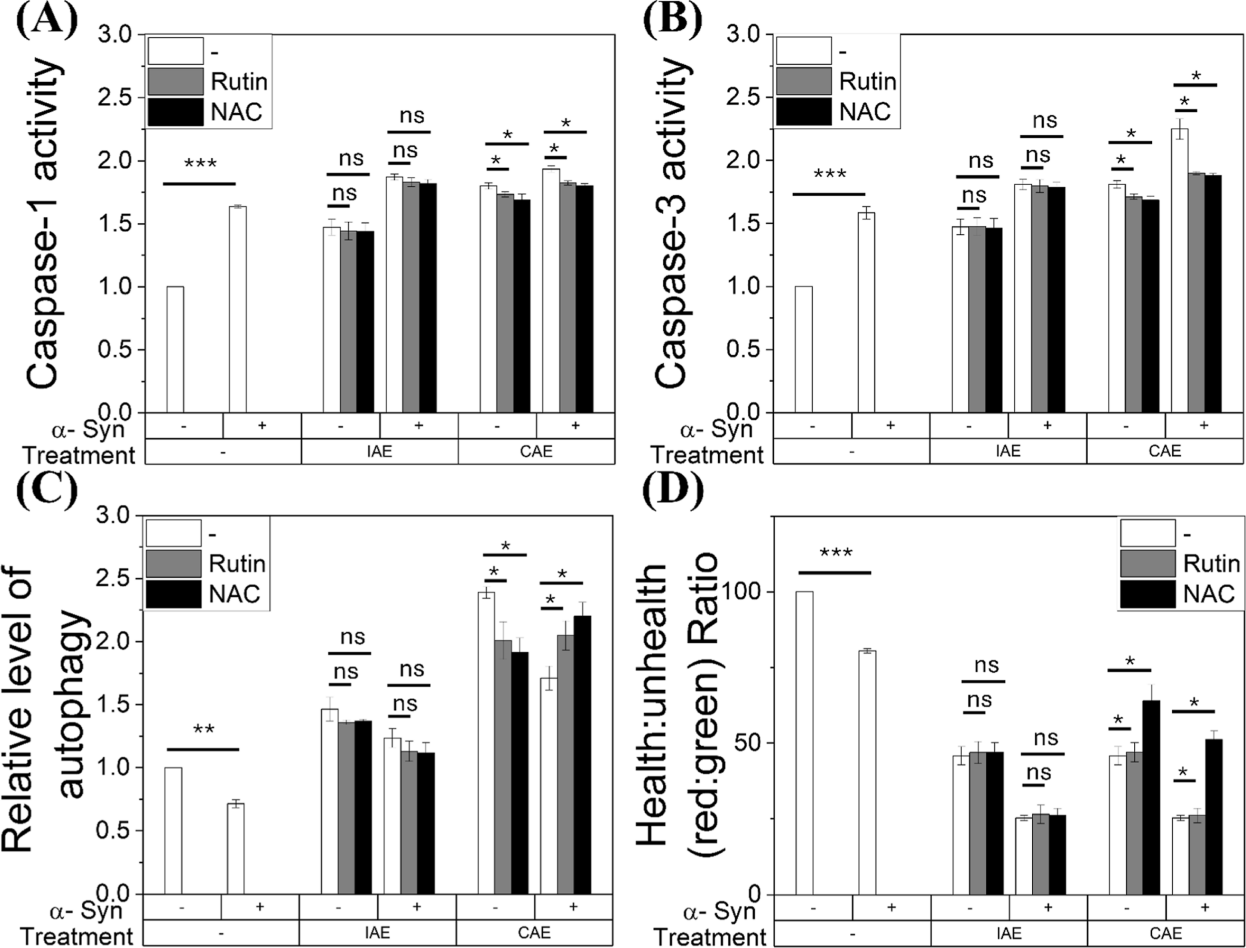
Effects of OP IAE and OP CAE on apoptosis, pyroptosis, autophagy, and mitochondrial membrane potential in SH-SY5Y cells with or without α-syn overexpression, and modulation by antioxidant cotreatment. SH-SY5Y cells, either control or overexpressing α-syn, were treated for 24 h with OP extracts in the presence or absence of cotreatment with rutin (0.2 mM) or NAC (0.5 mM). Cells were analyzed for (A) pyroptosis (caspase-1 activity), (B) apoptosis (caspase-3 activity), (C) autophagic activity, and (D) mitochondrial membrane potential polarization. Data are expressed as mean ± SD (*n* = 3). *p < 0.05, **p < 0.01, ***p < 0.001 versus the corresponding IAE- or CAE-only condition.

We further investigated whether antioxidant cotreatment could prevent the disruption of α-Syn homeostasis, a key pathological feature of synucleinopathies. In untreated SH-SY5Y cells overexpressing α-Syn, the protein was distributed diffusely throughout the cytosol (Figure 9A­(i)). Exposure to CAE promoted the formation of distinct, mesoscale α-Syn puncta, indicative of higher-order oligomers. Co-treatment with either Rutin or NAC substantially attenuated this phenotype, significantly reducing both the number and mean size of CAE-induced puncta (Figure 9A­(ii),B,C, *P* < 0.01 upon Rutin treatment and *P* < 0.001 up NAC treatment). This suggests that antioxidants can limit the oxidative stress–driven clustering of α-Syn into visible puncta. Conversely, distinct behavior was observed under IAE conditions. As established in Section 3.3, IAE treatment drives α-Syn toward a monomeric state without forming puncta; consequently, antioxidant cotreatment resulted in no observable change in α-Syn localization compared to IAE treatment alone (Figure 9A­(iii)). To determine if the reduction of visible puncta in CAE-treated cells corresponded to a restoration of the native protein state, we utilized FCS and FCCS. Despite the disappearance of visible puncta in CAE + Antioxidant conditions, quantitative analysis revealed no significant recovery of α-Syn diffusion coefficients or α-Syn−α-Syn interaction dynamics (Supporting Information Figure S7, *P* > 0.05, Table 5). Similarly, the monomeric shift induced by OP-IAE remained unaltered by antioxidant treatment (Table 6). Collectively, these results indicate that while Rutin and NAC can prevent the formation of large, toxic puncta in CAE-exposed cells, they are insufficient to restore the native oligomeric equilibrium disrupted by either pollutant.

**Figure 9:**
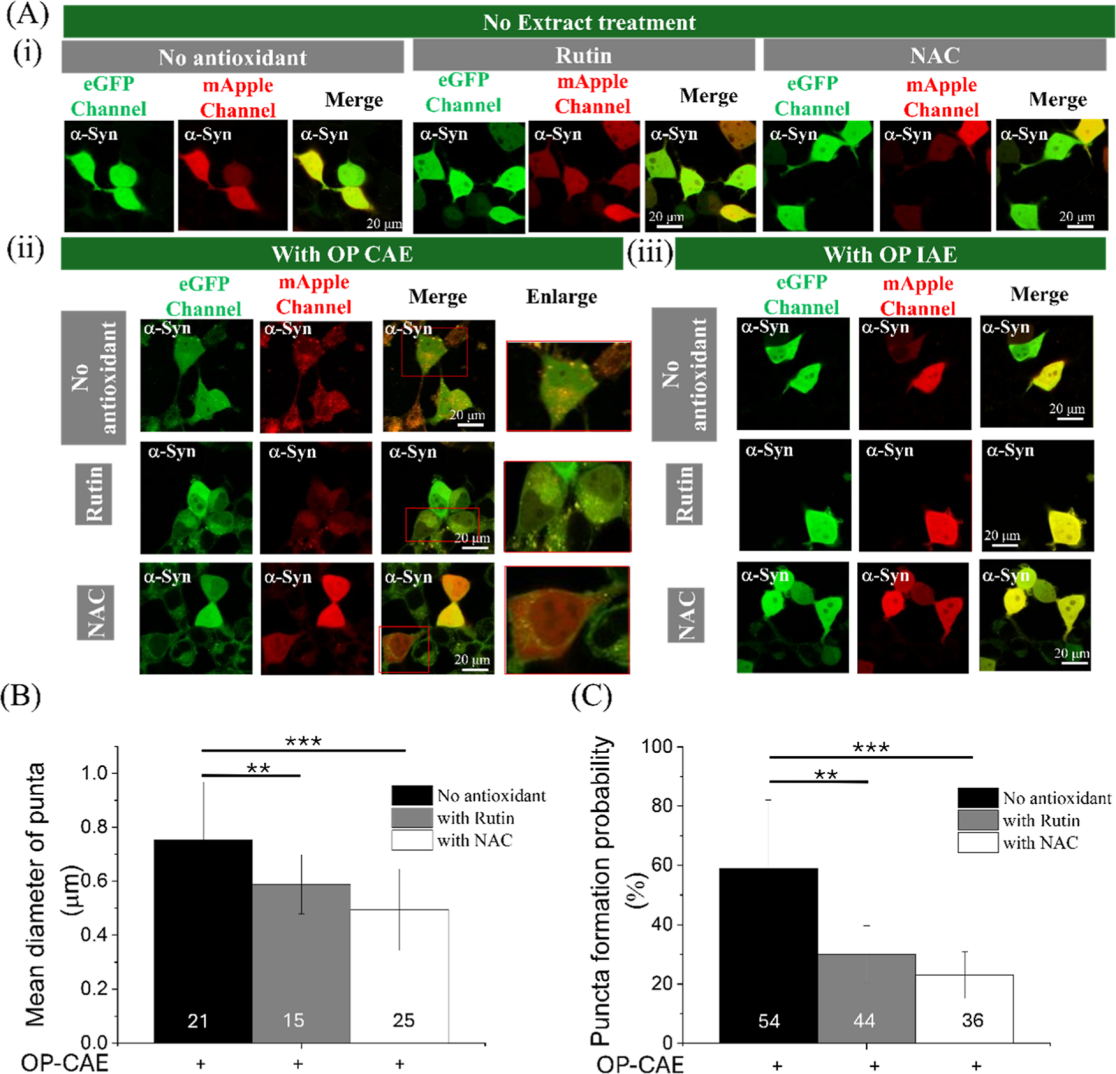
Effects of OP IAE and OP CAE and antioxidant treatment on α-syn oligomerization and puncta formation in living SH-SY5Y cells. (A) Representative confocal fluorescence images of SH-SY5Y cells coexpressing eGFP- and mApple-tagged α-syn, (i) no extract treatment and following 24 h treatment with (ii) OP IAE (0.18 μm–0.10 μm, type A incense) or (iii) OP CAE (0.56 μm–0.32 μm), in the presence or absence of cotreatment with rutin (0.2 mM) or NAC (0.5 mM). Scale bars: 40–50 μm as indicated. Insects highlight regions exhibiting α-syn puncta formation after OP CAE treatment. (B) Quantification of α-syn puncta size (observed only following OP CAE treatment) in the cytoplasm of individual SH-SY5Y cells and (C) puncta formation probability among investigated SH-SY5Y cells under the indicated conditions, as determined by image analysis of confocal sections. Numbers below the bars indicate the number of puncta analyzed for each group. Data are presented as mean ± SD.

**Table 5:** Diffusion Coefficients, Concentrations, and Anomalous Parameters of eGFP, mApple, eGFP−α-Syn, and mApple−α-Syn in SH-SY5Y Cells after 24 h of Transfection with OP CAE (0.56 μm–0.32 μm) with or without Antioxidative Reagent (Rutin and NAC)

	eGFP and mApple						
	no CAE	CAE		CAE + Rutin		CAE + NAC	
	(*N* = 3, *n* = 13)	type I (*N* = 5, *n* = 23)	type II (*N* = 5, *n* = 24)	type I (*N* = 4, *N* = 17)	type II (*N* = 4, *n* = 19)	type I (*N* = 6, *n* = 20)	type II (*N* = 3, *n* = 19)
diffusion coefficient (μm^2^/s) (free 3D diffusion fitting)	38.2 ± 4.1	22.1 ± 6.5	2.5 ± 1.7	20.1 ± 9.0	1.3 ± 1.9	21.5 ± 5.0	1.4 ± 1.2
eGFP concentration (nM)	300.5 ± 46.7	167.8 ± 101.4	267.4 ± 132.1	283.7 ± 189.4	381.5 ± 168.5	265.9 ± 238.3	425.4 ± 561.4
mApple concentration (nM)	299.7 ± 64.1	234.8 ± 133.3	258.5 ± 137.9	221.1 ± 159.5	322.1 ± 94.9	225.4 ± 107.3	360.3 ± 648.5
transport coefficient (μm^2^/s) (anomalous fitting)	41.6 ± 3.9 (α = 0.95 ± 0.06)	38.8 ± 8.5 (α = 0.74 ± 0.11)	29.7 ± 9.1 (α = 0.18 ± 0.11)	34.1 ± 4.1 (α = 0.71 ± 0.20)	29.3 ± 8.3 (α = 0.18 ± 0.11)	24.1 ± 8.0 (α = 0.72 ± 0.13)	11.8 ± 8.5 (α = 0.29 ± 0.16)
FCCS ratio (%)	23.6 ± 5.8	27.3 ± 8.4	25.5 ± 7.9	20.3 ± 9.8	19.7 ± 9.8	22.0 ± 7.7	19.3 ± 5.0

	eGFP−α-Syn and mApple−α-Syn						
	no CAE	CAE		CAE + Rutin		CAE + NAC	
	(*N* = 4, *n* = 21)	type I (*N* = 6, *n* = 23)	type II (*N* = 4, *n* = 19)	type I (*N* = 4, *n* = 17)	type II (*N* = 4, *n* = 14)	type I (*N* = 3, *n* = 14)	type II (*N* = 3, *n* = 13)
diffusion coefficient (μm^2^/s) (free 3D diffusion fitting)	23.6 ± 7.5	18.7 ± 4.4	1.2 ± 1.0	20.1 ± 5.9	0.7 ± 0.7	19.8 ± 8.0	1.1 ± 0.9
eGFP concentration (nM)	282.9 ± 97.6	278.5 ± 80.3	265.6 ± 144.7	363.1 ± 110.9	426.7 ± 308.0	313.1 ± 87.7	258.2 ± 76.7
mApple concentration (nM)	251.8 ± 118.0	286.6 ± 163.0	266.7 ± 96.9	280.6 ± 180.9	309.1 ± 54.5	246.8 ± 43.3	214.6 ± 65.0
transport coefficient (μm^2^/s) (anomalous fitting)	30.1 ± 7.1 (α = 0.81 ± 0.13)	35.2 ± 7.5 (α = 0.75 ± 0.10)	14.0 ± 9.7 (α = 0.19 ± 0.09)	33.1 ± 2.4 (α = 0.73 ± 0.26)	17.8 ± 1.2 (α = 0.19 ± 0.10)	33.0 ± 4.4 (α = 0.73 ± 0.09)	17.8 ± 1.2 (α = 0.24 ± 0.17)
FCCS ratio (%)	42.4 ± 5.6	20.9 ± 6.5	69.3 ± 14.1	20.5 ± 9.0	57.7 ± 9.0	17.5 ± 6.9	56.0 ± 12.9

**Table 6:** Diffusion Coefficients, Concentrations, and Anomalous Parameters of eGFP, mApple, eGFP-αSy, and mApple−α-Syn in SH-SY5Y Cells after 24 h of Transfection with OP IAE (0.18 μm–0.10 μm) with or without Antioxidative Reagent (Rutin and NAC)

	eGFP and mApple			
	no IAE (*N* = 3, *n* = 13)	IAE (*N* = 5, *n* = 17)	IAE + Rutin (*N* = 6, *N* = 19)	IAE + NAC (*N* = 3, *n* = 18)
diffusion coefficient (μm^2^/s) (free 3D diffusion fitting)	38.2 ± 4.1	28.7 ± 5.1	31.1 ± 5.8	28.8 ± 7.6
eGFP concentration (nM)	300.5 ± 46.7	295.6 ± 58.8	353.7 ± 86.7	358.8 ± 92.4
mApple concentration (nM)	299.7 ± 64.1	241.1 ± 36.4	291.2 ± 39.5	308.8 ± 40.1
transport coefficient (μm^2^/s) (anomalous fitting)	41.6 ± 3.9 (α = 0.95 ± 0.06)	37.1 ± 5.1 (α = 0.76 ± 0.09)	40.1 ± 11.6 (α = 0.73 ± 0.14)	36.2 ± 9.4 (α = 0.71 ± 0.10)
FCCS ratio (%)	23.6 ± 5.8	29.2 ± 8.3	25.8 ± 6.5	27.5 ± 8.2

	eGFP−α-Syn and mApple−α-Syn			
	no IAE (*N* = 4, *n* = 21)	IAE (*N* = 5, *n* = 17)	IAE + Rutin (*N* = 4, *n* = 18)	IAE + NAC (*N* = 3, *n* = 18)
diffusion coefficient (μm^2^/s) (free 3D diffusion fitting)	23.6 ± 7.5	20.1 ± 3.3	23.1 ± 7.6	23.7 ± 4.5
eGFP concentration (nM)	282.9 ± 97.6	343.4 ± 132.9	341.7 ± 83.3	288.0 ± 79.8
mApple concentration (nM)	251.8 ± 118.0	272.7 ± 74.4	282.4 ± 54.7	226.1 ± 42.5
transport coefficient (μm^2^/s) (anomalous fitting)	30.7 ± 7.7 (α = 0.81 ± 0.13)	37.9 ± 5.1 (α = 0.77 ± 0.13)	26.5 ± 6.5 (α = 0.71 ± 0.14)	33.1 ± \7.1 (α = 0.71 ± 0.07)
FCCS ratio (%)	42.4 ± 5.6	28.8 ± 8.0	26.2 ± 5.0	28.1 ± 8.4

## 4 Discussion

### 4.1 IAE-Induced Mitochondrial Dysfunction and Oxidative Stress

In our previous work, we demonstrated that IAEs elevate oxidative stress, disrupt mitochondrial membrane potential, and activate cell death programs.^26^ The robust intracellular accumulation of ROS we observed after IAE exposure is consistent with earlier reports showing that incense-derived PM elevates oxidant capacity and induces oxidative injury in airway and macrophage models.^62,64,66^ ROS can act both as proximal cytotoxic mediators and as amplifiers of mitochondrial dysfunction. Lipid-soluble combustion products—such as PAHs, quinones, and related organics—rapidly induce mitochondrial ROS generation, disrupt mitochondrial membrane potential, and drive bioenergetic failure.^89–92^ Mitochondrial depolarization directly couples to reduced ATP generation, activation of stress kinases, and increased vulnerability to PCD.^89,90^ Thus, the magnitude of mitochondrial membrane potential loss we observe with OP-IAE exposure is mechanistically expected, given the higher burden of membrane-active PAHs and quinones reported in incense-derived particles.^64,66^ Importantly, overexpression of α-syn further sensitized SH-SY5Y cells to oxidative stress and mitochondrial injury, consistent with prior evidence that α-syn interacts with mitochondrial import machinery, disrupts respiratory function, and amplifies ROS-driven damage.^93–95^ The toxicological signature of IAE exposure—marked ROS elevation, loss of mitochondrial membrane potential, activation of apoptosis and pyroptosis pathways, and impaired autophagy—is thus consistent with established paradigms of toxicant-induced neurodegeneration. Moreover, these effects are further amplified by α-Syn overexpression, reinforcing the mechanistic relevance of incense exposure as a potential environmental contributor to Parkinson’s disease pathology (Figure 10)

**Figure 10:**
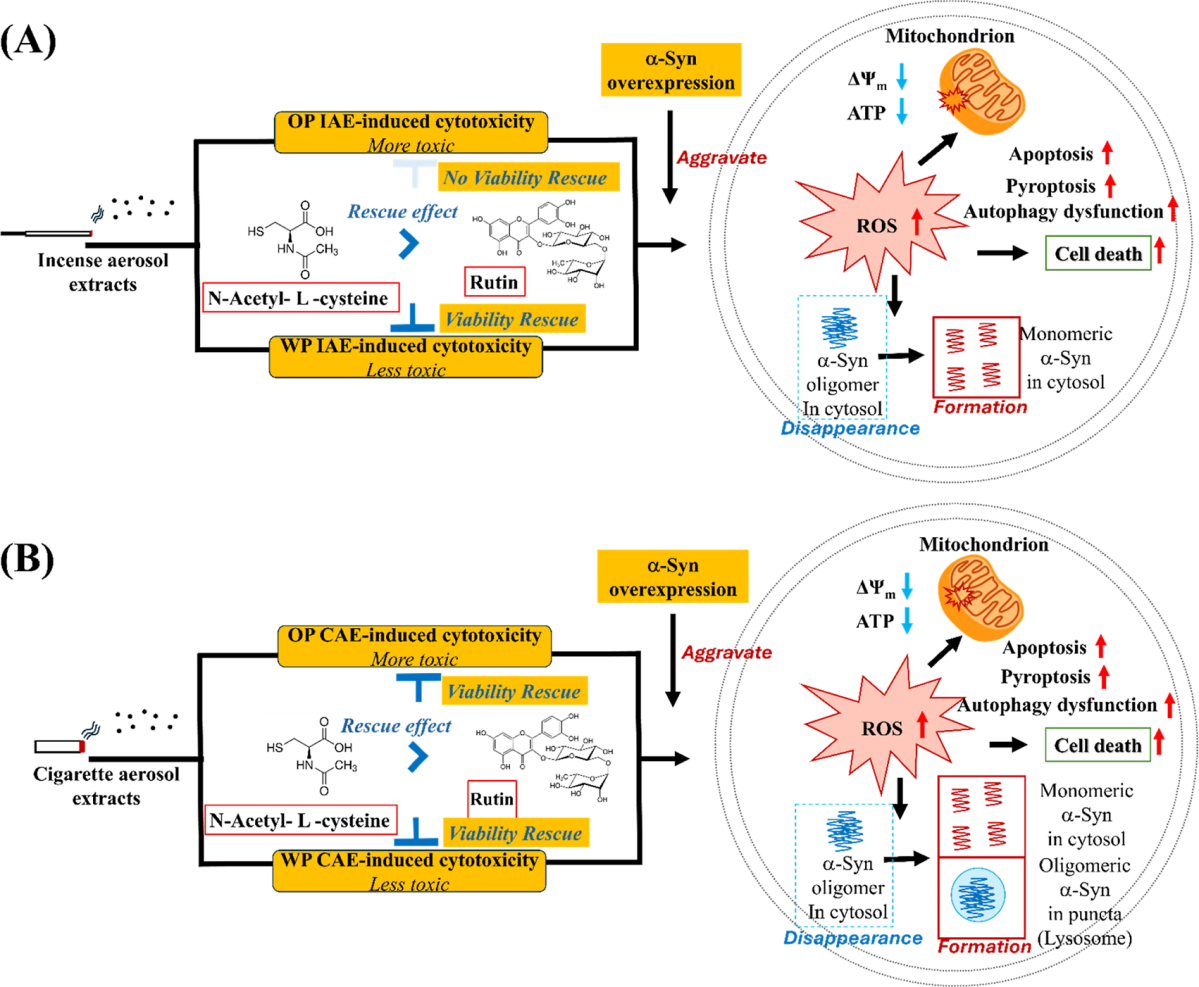
Differential cytotoxicity of IAE and CAE under α-syn overexpression. (A) IAE: OP fractions induce stronger cytotoxicity than WP fractions and exhibit limited responsiveness to antioxidant supplementation. Under α-syn overexpression, IAE exposure aggravates intracellular ROS generation, mitochondrial dysfunction, and activation of PCD pathways, accompanied by destabilization of α-syn organization characterized by loss of physiological oligomeric states without prominent puncta formation. (B) CAE: OP fractions show greater cytotoxicity than WP fractions; however, CAE-induced cellular stress, including PCD activation, remains markedly more responsive to antioxidant modulation. Under α-syn overexpression, CAE exposure induces ROS production, mitochondrial impairment, and PCD responses accompanied by α-syn puncta formation, which are attenuated by antioxidant treatment.

### 4.2 Distinct Effects of IAE on α-Syn Conformational Dynamics

A key finding of this study is the differential impact of incense versus cigarette aerosols on α-Syn homeostasis. It has been proposed that α-Syn principally exists as a folded helical tetramer^96^ and dynamically converts between tetramer and monomer.^97–101^ While familial PD–linked mutations destabilize this equilibrium,^100^ our data suggest environmental toxicants can mimic this pathogenic shift. Tetrameric α-syn is aggregation-resistant and nontoxic, whereas dissociation into monomers under oxidative stress or toxic exposures triggers cytotoxicity and promotes amyloid fibril formation.^98,100,102^ Our live-cell FCS experiments confirmed that wild-type α-syn in untreated cells predominantly exists in oligomeric tetrameric/hexameric forms, with diffuse cytosolic distribution and no detectable puncta.^43^ CAE, in contrast, strongly shifted α-Syn toward monomeric forms while simultaneously promoting lysosomal puncta formation (aggregates) and impairing autophagic flux.^39^ Interestingly, IAEs induced a distinct response: α-Syn equilibrium shifted exclusively toward monomeric species in the cytosol, without detectable puncta, even after 48 h of exposure (Figures 4 and 5). These findings reveal that IAE exposure destabilizes α-syn tetramers, favoring persistent monomerization rather than puncta formation. This observation is consistent with prior studies showing that environmental toxins, such as pesticides and airborne particulates, can shift the α-syn oligomerization equilibrium in a manner analogous to certain PD-linked genetic mutations.^39,103,104^ Monomeric α-syn has been reported to be more susceptible to oxidative modifications, such as methionine oxidation and tyrosine nitration, which enhance the formation of abnormal oligomeric or fibrillar species.^104^ These pathogenic species can bind mitochondrial membranes, further impairing mitochondrial potential and amplifying ROS production.^105,106^ Therefore, these results suggest that IAE acts as an environmental precipitant of PD-like pathology by simultaneously inducing oxidative stress, mitochondrial dysfunction, and dysregulation of apoptosis, pyroptosis, and autophagy, while also destabilizing α-syn structural homeostasis.

### 4.3 Divergent Antioxidant Rescue and Future Mechanistic Directions

We evaluated the protective effects of NAC and rutin on the cytotoxicity induced by both WP and OP fractions of IAE and CAE, and found a clear divergence based on the solvent-partitioned composition of the extracts. NAC—a hydrophilic thiol and glutathione precursor—provided significant protection against WP-IAE and both WP-CAE and OP-CAE, consistent with its well-established role in scavenging ROS and electrophiles in the aqueous cellular environment.^107^ Rutin, a polyphenolic flavonoid, exhibited moderate protection under WP-IAE and WP-CAE exposure, reflecting its limited cell permeability and partial hydrophilicity.^108^ Strikingly, neither NAC nor rutin conferred measurable protection against OP-IAE–induced cytotoxicity, suggesting that OP-IAE contains highly reactive or persistent toxins—such as quinones, high-molecular-weight PAHs, and other lipophilic electrophiles—that overwhelm traditional antioxidant defenses.^66^ This pronounced difference in antioxidant rescue efficacy likely stems from the distinct chemical composition of the extractable fractions: whereas CAE toxicity appears to be largely ROS-mediated and therefore responsive to antioxidant treatment, the OP-IAE fraction is enriched in membrane-permeable, lipophilic electrophiles and PAHs. These compounds may penetrate cellular and mitochondrial membranes, promote lipid peroxidation, and form covalent adducts with proteins—mechanisms that are largely inaccessible to hydrophilic antioxidants like NAC and rutin.^32,66^ As a result, the antioxidant-mediated rescue of CAE-induced α-syn puncta formation (Figure 9) and the recovery of cell viability (Figure 7) were evident with NAC/rutin. On the contrary, the persistent damage, pyroptotic/apoptotic signaling, and suppressed autophagic flux induced by OP-IAE were resistant to such intervention (Figure 8). These experimental results underscore the distinct toxicity profile of incense aerosols, especially their organic-phase components, and highlight the need for more comprehensive protective strategies beyond classical antioxidant therapy.

While the current study focuses on these comparative cellular outcomes, further in-depth investigation is required to fully elucidate this resistance mechanism. Future studies should employ comprehensive chemical profiling of organic-phase extracts using nontargeted LC–MS or GC–MS to identify specific electrophilic or redox-active constituents enriched in OP-IAE that underlie this resistance. Additionally, cellular assays targeting lipid peroxidation, protein carbonylation, and mitochondrial-specific ROS production will help distinguish ROS-dependent from ROS-independent injury mechanisms. Moreover, detailed analysis of α-Syn itself—such as oxidative post-translational modifications—would clarify how IAE uniquely destabilizes oligomerization equilibrium.

### 4.4 Experimental Relevance to Environmental Exposure

We acknowledge that the extract concentrations used in this in vitro study do not directly replicate instantaneous human inhalation exposure levels, which are influenced by deposition efficiency and clearance rates. Instead, the exposure system was intentionally designed to model accumulated cellular burden and hazard potential. This experimental strategy is widely adopted in aerosol toxicology to interrogate cellular stress responses and mechanistic vulnerability rather than to establish exposure–dose equivalence. The concentrations used here are consistent with prior studies of indoor combustion aerosols that induce similar mitochondrial dysfunction. Thus, our results define the cellular hazard and mechanistic susceptibility of neuronal cells to incense particulates, providing a platform to screen protective compounds, while recognizing that direct risk assessment would require integrated toxicokinetic modeling beyond the scope of this work.

## 5 Conclusion

Our findings indicate that incense smoke aerosols disrupt neuronal integrity through two synergistic mechanisms: (i) induction of oxidative injury and mitochondrial dysfunction, and (ii) destabilization of α-Syn oligomerization equilibrium toward pathogenic monomeric species. The organic fraction of incense aerosols is particularly potent which elicit oxidative stress and mitochondrial failure resistant to antioxidant rescue. In contrast, cigarette smoke toxicity is primarily ROS-driven and thus mitigable by NAC or rutin. Collectively, these data support the concept that incense exposure is an overlooked but significant environmental risk factor for Parkinson’s disease. By coupling classical oxidative injury with destabilization of α-syn conformers, incense aerosols mirror pathogenic mechanisms implicated in both genetic and environmental models of neurodegeneration. Given the global prevalence of incense burning, particularly in indoor environments with poor ventilation, our results underscore the need for stricter public health measures and further mechanistic investigation of incense smoke as a neurotoxicant.

## Supplementary Material



## Notes

Verification of α-syn transfection and fluorescence imaging under OP- and WP-IAE treatment (Figure S1); cytotoxicity of Rutin and NAC after 24 h exposure at 37 °C (Figure S2); Cell viability of SH-SY5Y cells with or without α-Syn expression after treatment with incense aerosol organic (OP) and water-phase (WP) extracts for 24 h at 37 °C, verified by MTT assay (Figure S3); confocal imaging and fluorescence (cross-)­correlation curves showing α-syn monomerization under incense aerosol exposure after 24 h (Figure S4) and 48 h (Figure S5); cell viability and antioxidant rescue profiles of SH-SY5Y cells (control or α-Syn overexpressing) cotreated with rutin or NAC under IAE/CAE exposure (Figure S6); and diffusion coefficients and synchronization ratios of α-syn in SH-SY5Y cells after IAE or CAE treatment in the presence or absence of antioxidants (Figure S7) (PDF)

## Abbreviations in Table

IAE: Incense aerosol extract
CAE: Cigarette aerosol extract
α-Syn: α-Synuclein
Type I: Homogeneous distribution region of α-Syn
Type: II Pancta region of α-Syn
FCCS: Fluorescence cross correlation spectroscopy
FCS: Fluorescence correlation spectroscopy
WT: Wild type
WP: Water-phase
OP: Organic-phase
PCD: Programmed cell death
a: Data is adapted from ref 26.
