# Supplementary material for: Incense Aerosol-Induced Neurotoxicity Disrupts α‑Synuclein Homeostasis in a Cellular Parkinson’s Disease Model, Distinct from Cigarette Aerosols
Source: Chem Res Toxicol. 2026 Mar 30;39(4):702–21. doi: 10.1021/acs.chemrestox.6c00091 (PMC13100986; doi:10.1021/acs.chemrestox.6c00091)
Supplement: Supplementary file 1 [file tx6c00091_si_001.pdf]

Supporting information

Incense Aerosol–Induced Neurotoxicity Disrupts α-Synuclein Homeostasis in a Cellular Parkinson's Disease Model, Distinct from Cigarette Aerosols

Yi-En Tseng[1,2,+], Ming-Chu Teng [1,2,+], Yu-Siou Huang[1,2,+], Chia-Hsuan Pan[2,3], Yuan-Pin Chang[2,3], Chia C. Wang[2,3], Hsiu-Fang Fan[1,2,3*]

[1] Institute of Medical Science and Technology, National Sun Yat-sen University, Kaohsiung, 804, Taiwan

[2] Department of Chemistry, National Sun Yat-sen University, Kaohsiung, 804, Taiwan

[3] Aerosol Science Research Center, National Sun Yat-sen University, Kaohsiung, 804, Taiwan

**** Correspondence can be sent to***
***HFF** (bendyfan@imst.nsysu.edu.tw)*

+ These authors have equal contributions to this work

Table of Contents

Figure S1. The bright field images and fluorescence images to verify the transfection efficiency of SH-SY5Y cells with expressing of eGFP-α-Syn and mApple-Syn after treatment of incense aerosols…………………………………………………………………………..S1

Figure S2. Cell viability of SH-SY5Y cells treated with incense aerosols……….…………………………………………………………………………..S2

Figure S3. Cell viability of SH-SY5Y cells with or without expressing of α-Syn after treatment of incense aerosol………..……………………………………………………………..S3

Figure S4. The confocal image, FCS and FCCS curves of SH-SY5Y cell treated with incense aerosol after 24 hours…………………………………………………………………..S4

Figure S5. The confocal image, FCS and FCCS curves of SH-SY5Y cell treated with incense aerosol after 24 hours…………………………………………………………………..S5

Figure S6. Cytotoxicity curves used for IC_{50} determination…………………………………………………………………………………..S6

Figure S7. Diffusion coefficient and synchronization ratio analysis……….………………………………………………………………………..S7

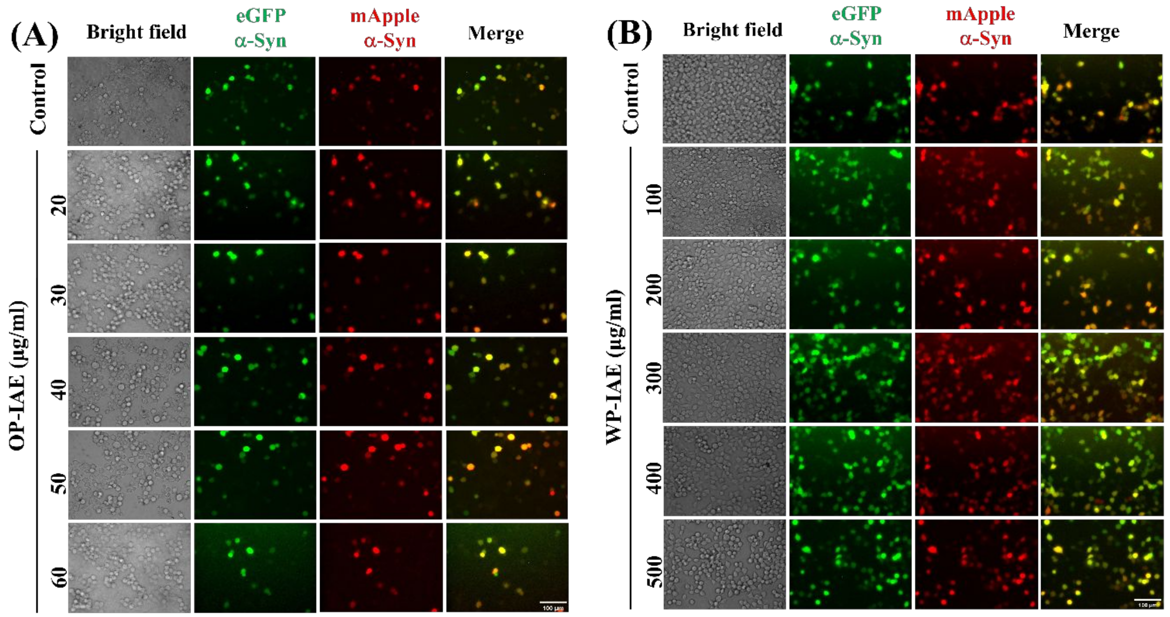

Figure S1 The bright field images and fluorescence images to verify the transfection efficiency of SH-SY5Y cells with expressing of eGFP-α-Syn and mApple-Syn after treatment of (A) Type A (sandalwood dominant) OP-IAE in various concentrations, (B) Type A (sandalwood dominant) WP-IAE in various concentrations.

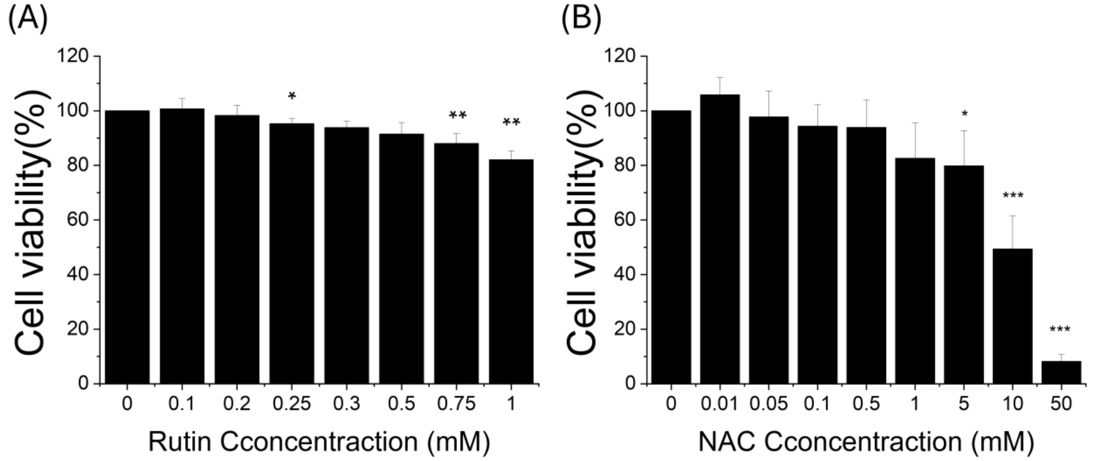

Figure S2. Cell viability of SH-SY5Y cells treated with (A) Rutin and (B) NAC for 24 h at 37 °C, as determined by the MTT assay. *Statistical significance versus no NAC treatment: Paired t-test, *$p < 0.05$, **$p < 0.01$, ***$p < 0.001$.

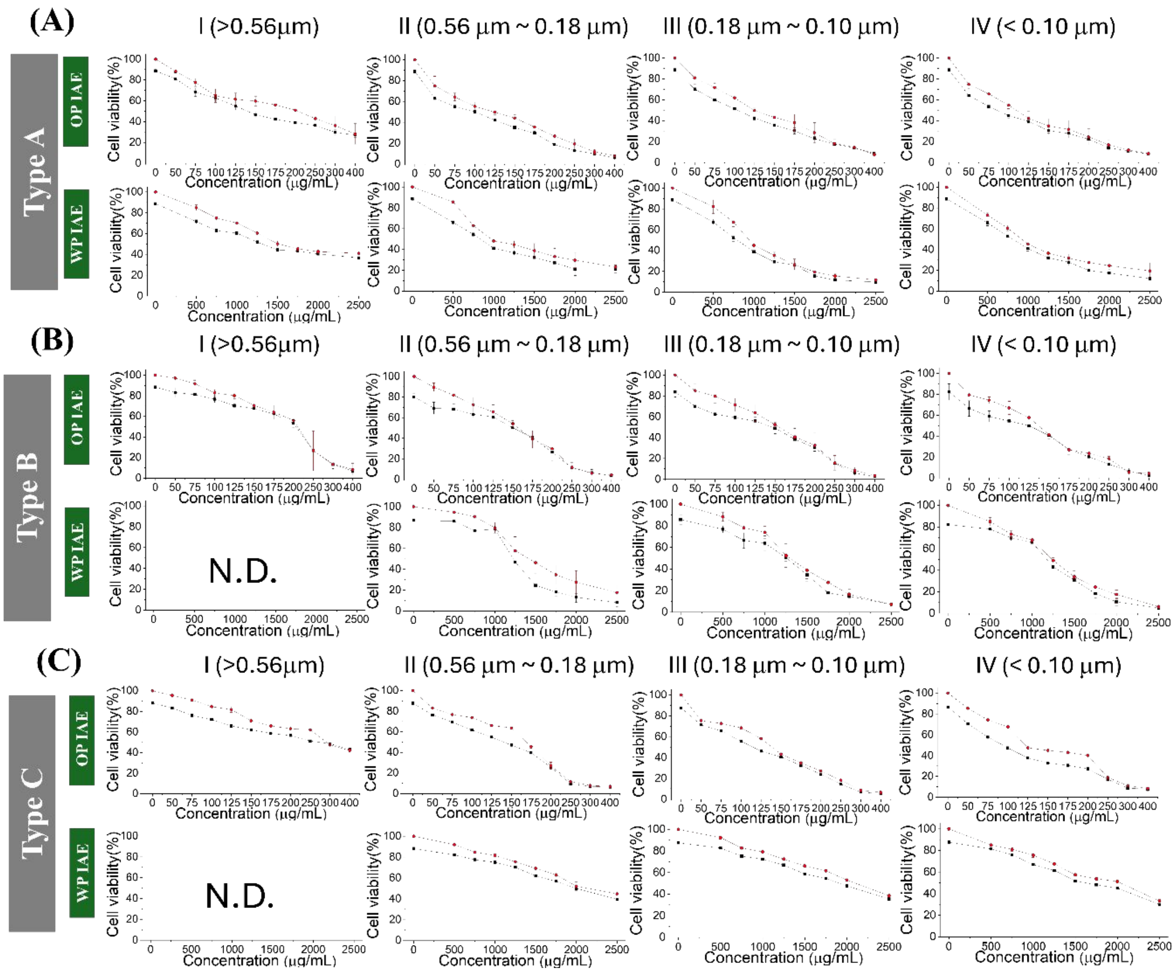

Figure S3 Cell viability of SH-SY5Y cells with or without expressing of α-Syn after treatment of incense aerosol (A) OP (B) WP extraction for 24 h at 37°C verified by MTT assay. N (repeat of experiment) is 3 for each condition. I~VI indicates the size of incense aerosol obtained with MOUDI.

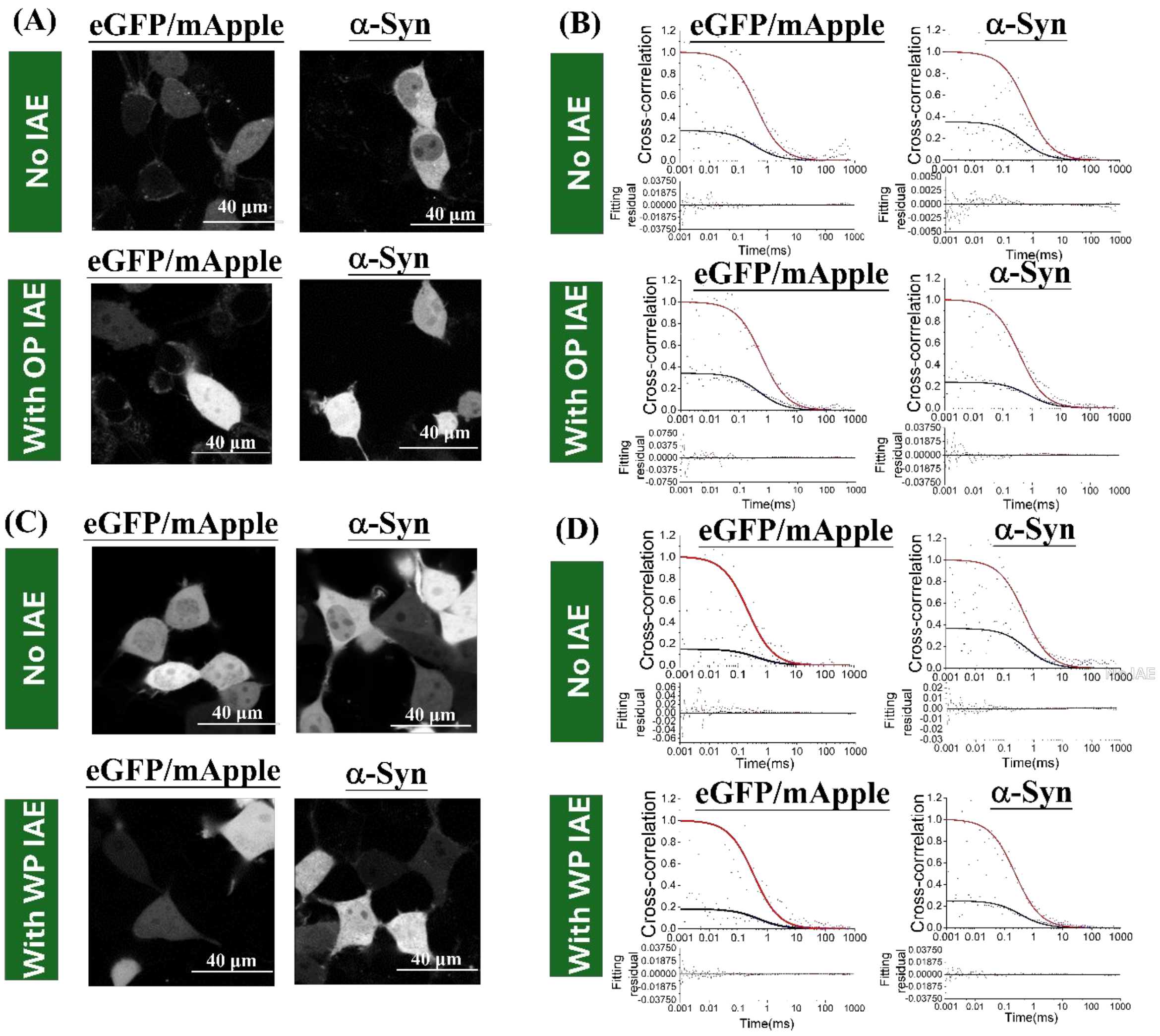

Figure S4. (A) The confocal image and (B) FCS curve and normalized FCCS curve of SH-SY5Y cells co-expressing eGFP-α Syn and mApple-α Syn with or without treatment of OP IAE for 24 h at 37°C. (C) The confocal image and (D) FCS curve and normalized FCCS curve of SH-SY5Y cells co-expressing eGFP-α Syn and mApple-α Syn with or without treatment of WP IAE for 24 h at 37°C.

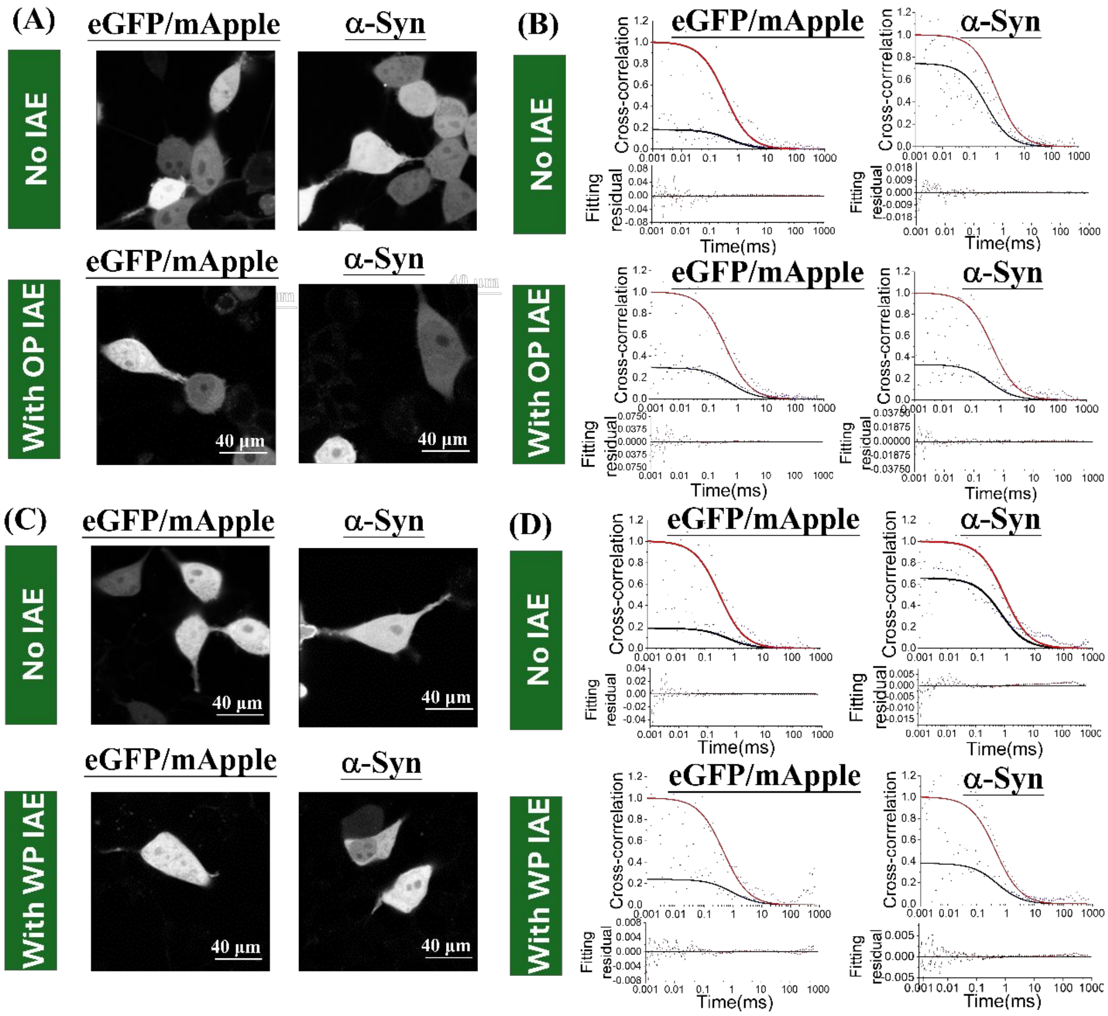

Figure S5. (A) The confocal image and (B) FCS curve and normalized FCCS curve of SH-SY5Y cells co-expressing eGFP-α Syn and mApple-α Syn with or without treatment of OP IAE for 48 h at 37°C. (C) The confocal image and (D) FCS curve and normalized FCCS curve of SH-SY5Y cells co-expressing eGFP-α Syn and mApple-α Syn with or without treatment of WP IAE for 48 h at 37°C.

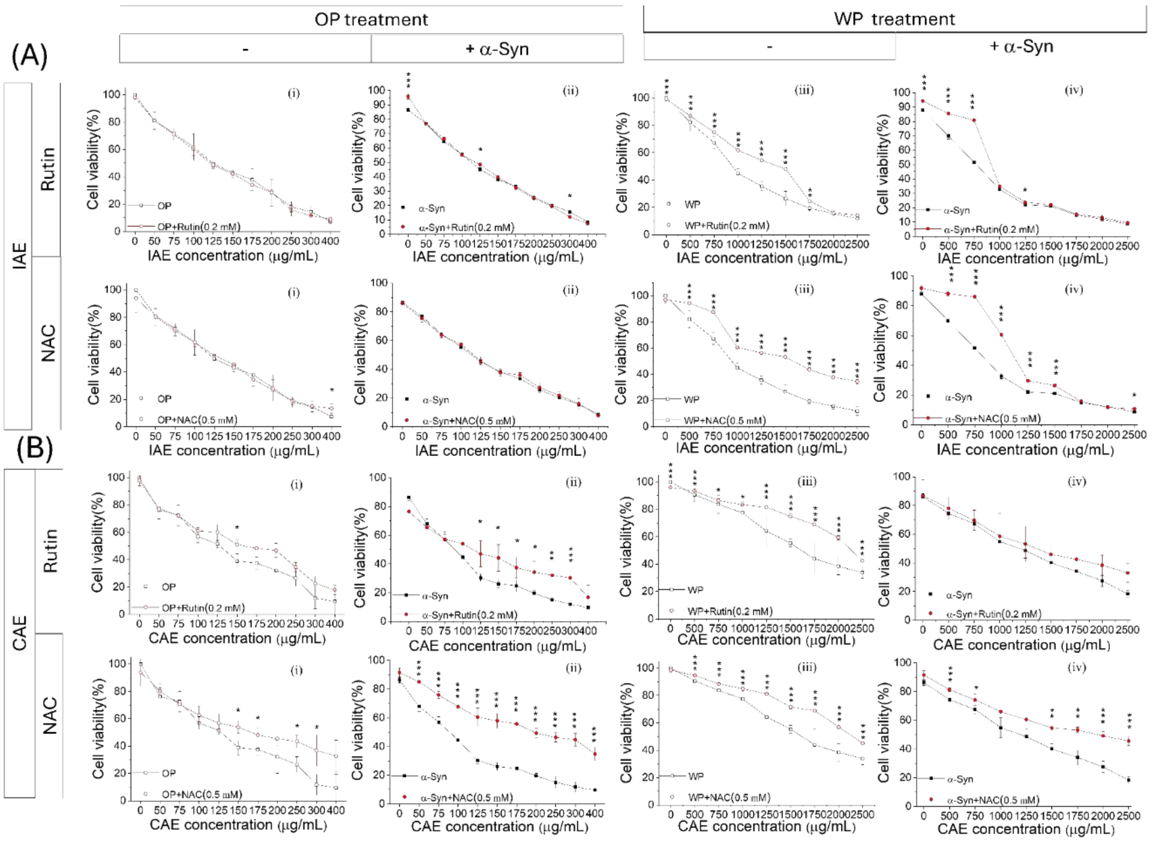

Figure S6. MTT assay results showing the viability of SH-SY5Y cells (control or α-syn-overexpressing) after 24 h exposure to (A) IAEs (0.18 μm ~0.10 μm, most abundant fraction from Type A incense) or (B) CAEs (0.56 μm ~0.32 μm, most abundant fraction), with or without co-treatment with rutin (0.2 mM) or NAC (0.5 mM). Data are expressed as the percentage of untreated controls (mean ± SD, n = 3). *Statistical significance versus IAE or CAE alone: Paired t-test, *$p < 0.05$, **$p < 0.01$, ***$p < 0.001$.

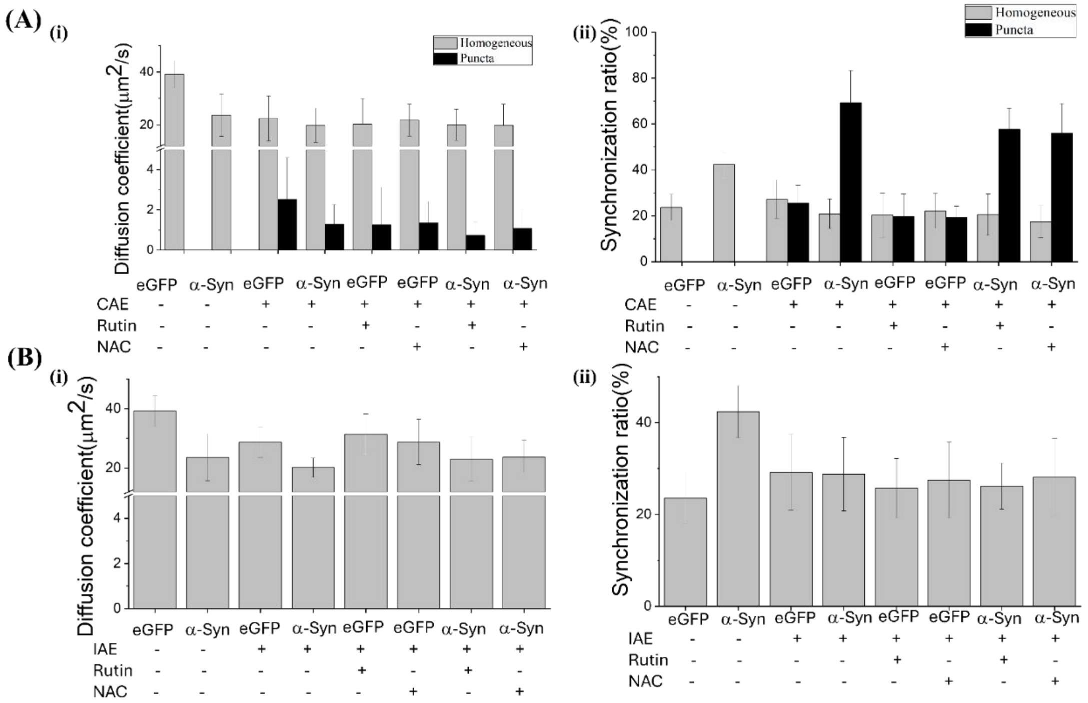

Figure S7. SH-SY5Y cells co-expressing eGFP–α-syn and mApple–α-syn were treated for 24 h with OP IAE (0.18 μm ~0.10 μm, most abundant fraction from Type A incense) or OP CAE (0.56 μm ~0.32 μm, most abundant fraction), in the presence or absence of rutin (0.2 mM) or NAC (0.5 mM). (A, C) Diffusion coefficients and (B, D) synchronization ratios were determined by fluorescence cross-correlation spectroscopy (FCCS) in the cytoplasm of individual cells. Data are shown as mean ± SD. *Statistical significance versus IAE or CAE alone: Welch's t-test, #, $0.05 < p < 0.1$*$p < 0.05$, **$p < 0.01$, ***$p < 0.001$
